# Precursor-Directed Combinatorial Biosynthesis of Cinnamoyl, Dihydrocinnamoyl, and Benzoyl Anthranilates in *Saccharomyces cerevisiae*

**DOI:** 10.1371/journal.pone.0138972

**Published:** 2015-10-02

**Authors:** Aymerick Eudes, Veronica Teixeira Benites, George Wang, Edward E. K. Baidoo, Taek Soon Lee, Jay D. Keasling, Dominique Loqué

**Affiliations:** 1 Joint BioEnergy Institute, Emery Station East, 5885 Hollis St, 4^th^ Floor, Emeryville, California, 94608, United States of America; 2 Physical Biosciences Division, Lawrence Berkeley National Laboratory, Berkeley, California, 94720, United States of America; 3 Graduate Program, San Francisco State University, San Francisco, California, 94132, United States of America; 4 Department of Bioengineering & Department of Chemical & Biomolecular Engineering, University of California, Berkeley, California, 94720, United States of America; University of Copenhagen, DENMARK

## Abstract

Biological synthesis of pharmaceuticals and biochemicals offers an environmentally friendly alternative to conventional chemical synthesis. These alternative methods require the design of metabolic pathways and the identification of enzymes exhibiting adequate activities. Cinnamoyl, dihydrocinnamoyl, and benzoyl anthranilates are natural metabolites which possess beneficial activities for human health, and the search is expanding for novel derivatives that might have enhanced biological activity. For example, biosynthesis in *Dianthus caryophyllus* is catalyzed by hydroxycinnamoyl/benzoyl-CoA:anthranilate *N*-hydroxycinnamoyl/ benzoyltransferase (HCBT), which couples hydroxycinnamoyl-CoAs and benzoyl-CoAs to anthranilate. We recently demonstrated the potential of using yeast (*Saccharomyces cerevisiae*) for the biological production of a few cinnamoyl anthranilates by heterologous co-expression of 4-coumaroyl:CoA ligase from *Arabidopsis thaliana* (4CL5) and HCBT. Here we report that, by exploiting the substrate flexibility of both 4CL5 and HCBT, we achieved rapid biosynthesis of more than 160 cinnamoyl, dihydrocinnamoyl, and benzoyl anthranilates in yeast upon feeding with both natural and non-natural cinnamates, dihydrocinnamates, benzoates, and anthranilates. Our results demonstrate the use of enzyme promiscuity in biological synthesis to achieve high chemical diversity within a defined class of molecules. This work also points to the potential for the combinatorial biosynthesis of diverse and valuable cinnamoylated, dihydrocinnamoylated, and benzoylated products by using the versatile biological enzyme 4CL5 along with characterized cinnamoyl-CoA- and benzoyl-CoA-utilizing transferases.

## Introduction

**Fig 1:**
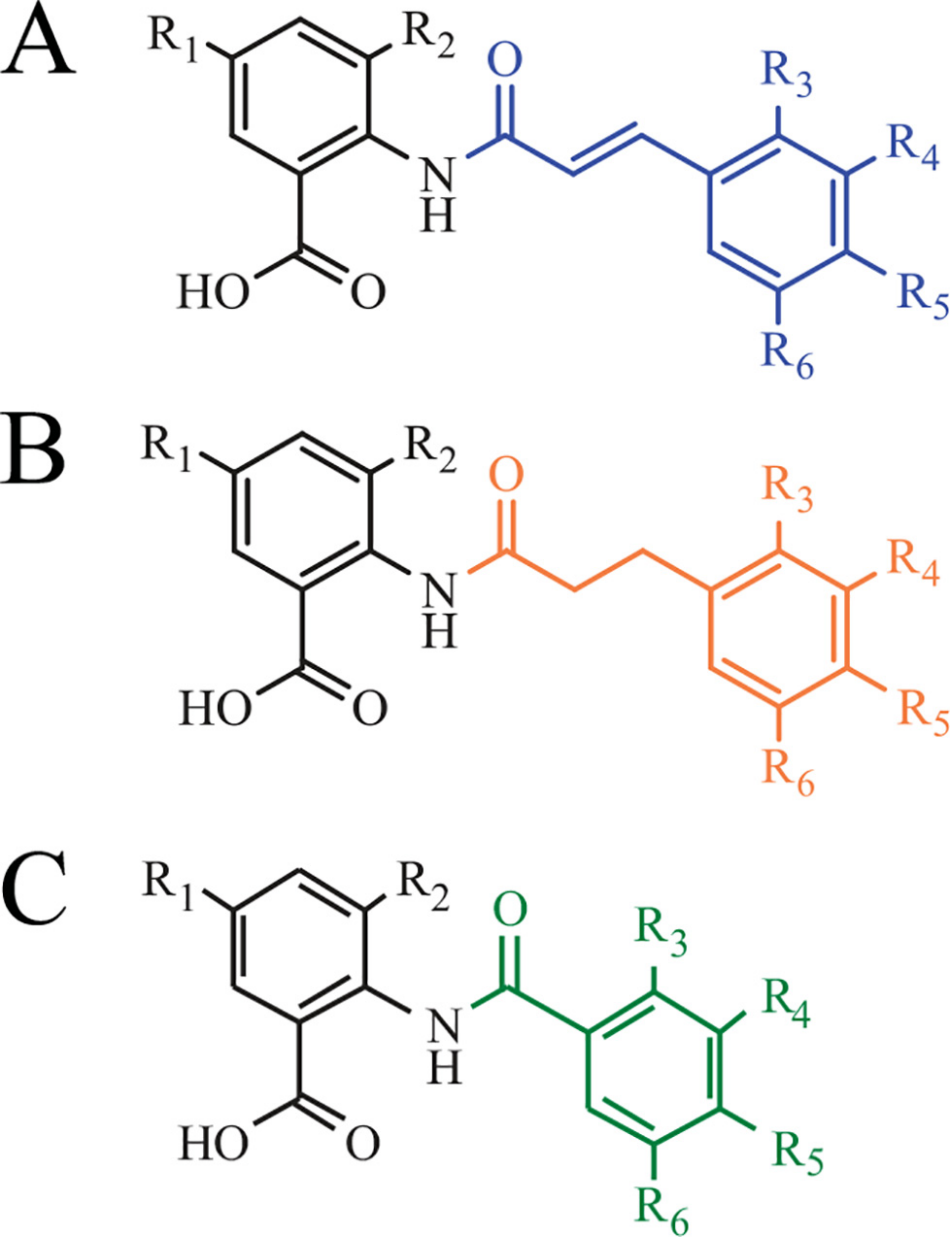
Structure of cinnamoyl, dihydrocinnamoyl, and benzoyl anthranilates. **(A)** Cinnamoyl anthranilates. Tranilast: R_1_ = R_2_ = R_3_ = R_6_ = H, R_4_ = R_5_ = OMe. **(B)** Dihydrocinnamoyl anthranilates. DHavnD: R_1_ = R_2_ = R_3_ = R_4_ = R_6_ = H, R_5_ = OH. **(C)** Benzoyl anthranilates. Dianthramide B from *D*. *caryophyllus*: R_1-6_ = H.

Cinnamoyl and benzoyl anthranilates are bipartite molecules consisting of cinnamate or benzoate moieties amide-linked to anthranilic acids (Fig 1). The beneficial pharmacological effects of these molecules on human health have been well-documented over the past few years. For example, avenanthramides are natural cinnamoyl anthranilates found in oats and possess antioxidant, anti-inflammatory, and antiproliferative bioactivities [1,2]. Tranilast ([*N*-(3’,4’-dimethoxycinnamoyl)-anthranilic acid], Fig 1A) is a synthetic cinnamoyl anthranilate marketed in Japan for the treatment of allergic diseases, scleroderma, and hypertrophic scars associated with excessive fibrotic response [3]. In particular, tranilast is an antifibrotic agent that inhibits several profibrotic growth factors [4–6]. Recent efforts have been made for the development of tranilast analogs to optimize the antifibrotic effects and reduce toxicity at higher doses [7]. For instance, modification of functional groups on the cinnamoyl ring and the introduction of halogens resulted in cinnamoyl anthranilates with higher bioavailability and enhanced inhibitory effects on fibrosis [8–12]. Other structure optimizations have included double bond saturation resulting in dihydrocinnamoyl anthranilates such as dihydroavenanthramide D (DHavnD, Fig 1B), which is an anti-inflammatory used for the treatment of skin disorders and is currently evaluated for its antidiabetic and anticancer effects [13–15]. Benzoyl anthranilates (Fig 1C) are found in some plant species such as *D*. *caryophyllus* [16]; and several analogs were shown to inhibit human aldo-keto reductases involved in different pathophysiological conditions such as prostate cancer [17], as well as to possess cytotoxic activity toward cancer cell lines [18]. Moreover, certain halogenated benzoyl anthranilates are candidates for the treatment of infectious diseases because of their inhibitory effects on the malaria agent *Plasmodium falciparum* [19], the human African trypanosomiasis agent *Trypanosoma brucei* [20,21], and the opportunistic pathogenic bacterium *Pseudomonas aeruginosa* [22,23].

**Fig 2:**
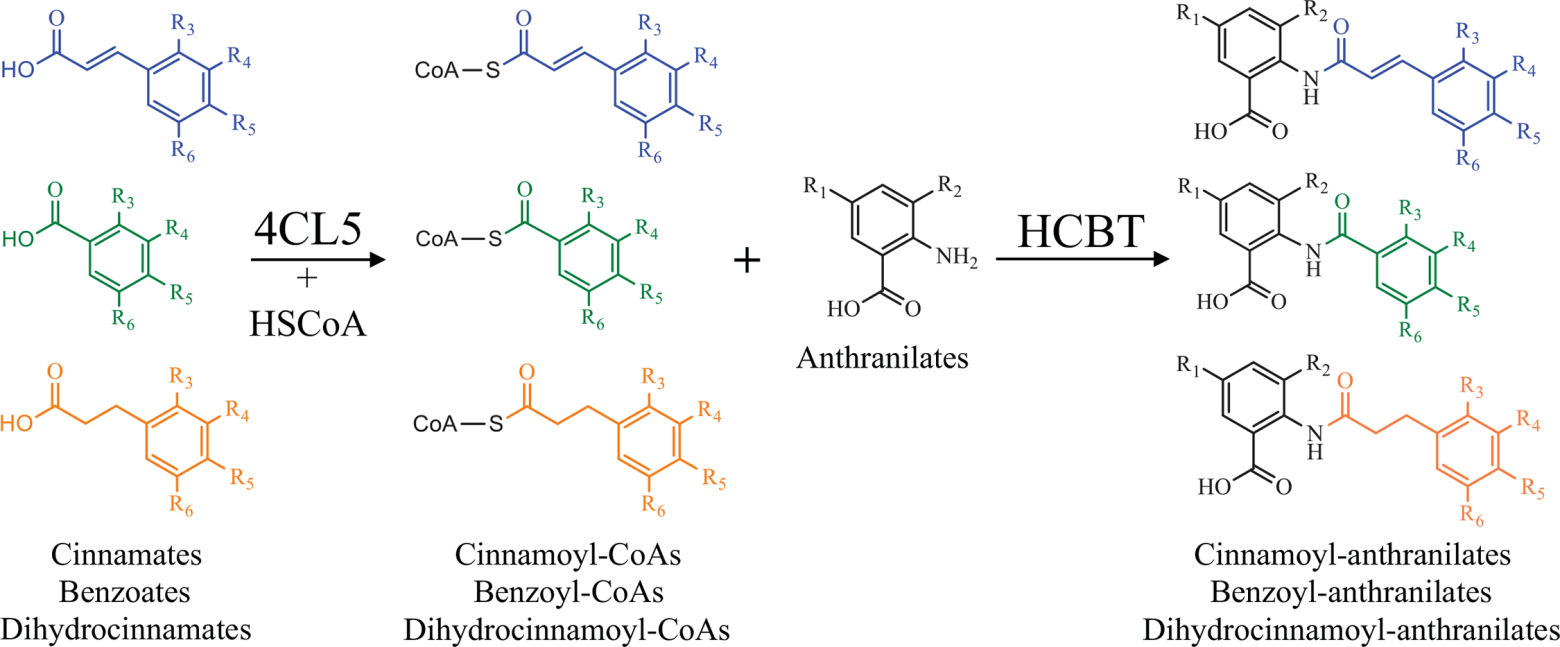
Strategy used for the biological synthesis of cinnamoyl, dihydrocinnamoyl, and benzoyl anthranilates. Diagram of the reactions catalyzed by 4CL5 and HCBT in the yeast strain engineered for the production of various cinnamoyl, dihydrocinnamoyl, and benzoyl anthranilates upon feeding with cinnamates, dihydrocinnamates, or benzoates (donors); and with anthranilates (acceptors). HSCoA, Coenzyme A.

The chemical synthesis of pharmaceuticals such as cinnamoyl and benzoyl anthranilates—or their purification from source organisms—consumes nonrenewable petroleum-based chemicals, generates toxic byproducts that require downstream waste-processing, and increases production costs. By contrast, biological synthesis is an eco-friendly production method with reduced requirements for toxic chemicals and natural resources. It offers consistent quality, scalability, simple extraction, and potential for higher synthesis efficiency [24]. In addition, biological synthesis could expand the chemical diversity of natural products, the structural complexity of which is sometimes challenging to achieve using multistep chemical synthesis [25]. In this area, the industrial microorganism yeast (*Saccharomyces cerevisiae*) has emerged as a powerful tool for the biosynthesis of secondary metabolites considering its advantages for the expression of complex metabolic pathways [26]. We previously reported on a yeast strain engineered for the production of tranilast and several analogs [27]. Cinnamates supplied to this strain are converted into coumaroyl-CoAs by 4-coumaroyl:CoA ligase 5 (4CL5) from *Arabidopsis thaliana* and coupled to anthranilate or 3-hydroxyanthranilate by hydroxycinnamoyl/benzoyl-CoA:anthranilate *N*-hydroxycinnamoyl/ benzoyltransferase (HCBT) from *D*. *caryophyllus* (Fig 2). In an earlier study, 13 methoxylated and hydroxylated cinnamates were successfully used as precursors for the production of the corresponding hydroxy/methoxycinnamoyl anthranilates [27]. Here, we show how we extended our yeast production platform by screening several new cinnamate derivatives that could potentially be converted by our yeast strain into cinnamoyl anthranilates and explored benzoates as precursors for the production of benzoyl anthranilates (Fig 2). First, a series of halogenated cinnamates were tested because of the importance of halogen groups—particularly fluoride—in drug development [28,29]. Second, several dihydrocinnamates, which correspond to cinnamates with a saturated double bond on the propanoid tail, were tested and successfully converted into dihydrocinnamoyl anthranilates—including those that were halogenated. Third, since HCBT is known to use benzoyl-CoA in addition to coumaroyl-CoA [30], we attempted to feed the yeast strain with benzoic acid derivatives and confirmed production of a series of halogenated benzoyl anthranilates.

Altogether, our data demonstrate that the substrate promiscuity of both 4CL5 and HCBT can be exploited for biological synthesis of structurally diverse cinnamoyl, dihydrocinnamoyl, and benzoyl anthranilates of potential pharmaceutical value.

## Materials and Methods

### Chemicals

The cinnamates, dihydrocinnamates (or 3-phenylpropionates), and benzoates used for the yeast feeding experiments are listed in S1, S2 and S3 Tables and were purchased from VWR International (Radnor, PA, USA). DHavnD and dianthramide B were obtained from Enamine Ltd (Monmouth Jct., NJ) and Sigma-Aldrich (Saint-Louis, MO), respectively.

### Expression of 4CL5 and HCBT in yeast

The pDRf1-4CL5-HCBT1, pDRf1-HCBT1, and pDRf1-4CL5 vectors [27] were used for the expression of *At4CL5* (*At3g21230*, also named *At4CL4* in original studies [31]) and a codon-optimized *HCBT* (GenBank: Z84385.1) under the control of the constitutive promoters *P*_*HXT7*_ and *P*_*PMA1*_, respectively. The *S*. *cerevisiae pad1* knockout (*MAT*a *his3*∆*1 leu2*∆*0 met15*∆*0 ura3*∆*0* ∆*pad1*, ATCC 4005833) [32] was transformed using the Frozen-EZ Yeast Transformation II Kit™ (Zymo Research Corporation, Irvine, CA) and selected on solid medium containing Yeast Nitrogen Base (YNB) without amino acids (Difco 291940; Difco, Detroit, MI) supplemented with 3% glucose and 1X dropout-uracil (CSM-ura; Sunrise Science Products, San Diego, CA). A *pad1* knockout was chosen because PAD1 is a known phenylacrylic acid decarboxylase whose deletion in yeast prevents the degradation of exogenously supplied cinnamates [33, 34].

### Production of cinnamoyl, dihydrocinnamoyl, and benzoyl anthranilates

An overnight culture from a single colony of the pDRf1-4CL5-HCBT1 recombinant yeast grown on 2X YNB medium without amino acids, supplemented with 6% glucose and 2X CSM-Ura, was used to inoculated 4 mL of fresh minimal medium at an OD_600_ = 0.15 and shaken at 200 rpm at 30°C. All precursors were prepared in DMSO and added 5 hours post inoculation at the concentrations indicated in S1, S2 and S3 Tables. The anthranilate acceptors were added to the medium at a final concentration of 300 μM (for anthranilate, 3-hydroxyanthranilate, 3-methylanthranilate, and 5-nitroanthranilate) or 50 μM (for 3-chloroanthranilate, 5-methylanthranilate, 3-methoxyanthranilate, 5-fluoroanthranilate, 5-iodoanthranilate, and 5-chloroanthranilate). These concentrations were selected to limit toxicity and growth inhibition due to either the supplied precursors or the metabolites produced. The cultures were shaken at 200 rpm at 30°C for 24 h in the presence of the precursors for the production of cinnamoyl, dihydrocinnamoyl, and benzoyl anthranilates. Yeast colonies harboring the pDRf1-HCBT1 or pDRf1-4CL5 control vectors were grown under similar conditions. For the detection of metabolites, an aliquot of the culture medium was collected and cleared by centrifugation (21,000x*g* for 5 min at 4°C), mixed with an equal volume of cold methanol:water (1:1, v/v), and filtered using Amicon Ultra centrifugal filters (3,000 Da MW cutoff regenerated cellulose membrane; Millipore, Billerica, MA) prior to LC-TOF MS analysis. The separation and identification of the metabolites were performed using high-performance liquid chromatography (HPLC), electrospray ionization (ESI), and time-of-flight (TOF) mass spectrometry (MS) as previously described [35]. For each compound, the measured masses agreed with the expected theoretical masses within less than 5 ppm mass error. Standard solutions of DHavnD and dianthramide B were prepared in methanol:water (1:1, v/v). Values obtained for the production of DHavnD and dianthramide B are the average of four replicates (*n* = 4). ESI-MS spectra of other cinnamoyl, dihydrocinnamoyl, and benzoyl anthranilates were obtained from single feeding experiments for each combination of precursors.

## Results

### Production of halogenated cinnamoyl anthranilates

A yeast strain that co-expresses 4CL5 and HCBT was used as a catalyst for the production of non-natural halogenated cinnamoyl anthranilates. We showed previously that HCBT can accept anthranilate or 3-hydroxyanthranilate as substrates for the production of cinnamoyl anthranilates [27]. We further investigated the substrate promiscuity of HCBT and the possibility of producing additional cinnamoyl conjugates by feeding the yeast strain with new anthranilates in combination with *p*-coumarate. Of 10 anthranilates individually supplied to the culture medium, five novel *p*-coumaroyl anthranilates were conclusively produced upon feeding with 3-methylanthranilate, 3-methoxyanthranilate, 3-chloroanthranilate, 5-methylanthranilate, and 5-fluoroanthranilate—indicating that HCBT can also accept these anthranilate analogs (Table 1, S1 Fig). Based on their expected masses, these compounds were identified by LC-MS analysis of the culture medium but could not be detected in control yeast cultures grown with only anthranilates (without *p*-coumarate). Next, to assess the capacity of the yeast strain to produce non-natural cinnamoyl anthranilates, we fed the 4CL5- and HCBT-expressing yeast strain several halogenated cinnamates in combination with the seven different anthranilates identified as HCBT acceptors. As a result, 45 novel halogenated cinnamoyl anthranilates were biosynthesized out of 98 combinations tested using a series of 14 fluorinated, chlorinated, and brominated cinnamates (Table 1, S1 Fig). These results demonstrate the coenzyme A-ligase activity of 4CL5 toward these non-natural cinnamates and the capacity of HCBT to couple the corresponding CoA-thioesters to various anthranilates.

**Table 1:** Structural characteristics of the cinnamoyl anthranilates (general structure shown in Fig 1A) produced in yeast and their identification based on dominant ion masses in ESI-MS spectra. Values were obtained from single feeding experiments for each combination of precursors.

Donor	Acceptor	Cinnamoyl anthranilates	R_1_	R_2_	R_3_	R_4_	R_5_	R_6_	Formula	Theoretical mass [M-H]^-^	Measured mass [M-H]^-^	Mass accuracy^a^ (ppm)	Retention time (min)	Mass spectrum # in S1 Fig
*p*-coumaric acid	5-methylanthranilic acid	*N*-(4’-hydroxycinnamoyl)-5-methylanthranilic acid	CH_3_	H	H	H	OH	H	C_17_H_15_NO_4_	296.0928	296.0937	-3.04	12.56	1
*p*-coumaric acid	3-methylanthranilic acid	*N*-(4’-hydroxycinnamoyl)-3-methylanthranilic acid	H	CH_3_	H	H	OH	H	C_17_H_15_NO_4_	296.0928	296.0925	1.01	10.95	2
*p*-coumaric acid	5-fluoroanthranilic acid	*N*-(4’-hydroxycinnamoyl)-5-fluoroanthranilic acid	F	H	H	H	OH	H	C_16_H_12_FNO_4_	300.0678	300.0678	0.00	11.05	3
*p*-coumaric acid	3-methoxyanthranilic acid	*N*-(4’-hydroxycinnamoyl)-3-methoxyanthranilic acid	H	OCH_3_	H	H	OH	H	C_17_H_15_NO_5_	312.0877	312.0874	0.96	9.99	4
*p*-coumaric acid	3-chloroanthranilic acid	*N*-(4’-hydroxycinnamoyl)-3-chloroanthranilic acid	H	Cl	H	H	OH	H	C_16_H_12_ClNO_4_	316.0382	316.0383	-0.32	10.46	5
2-fluorocinnamic acid	anthranilic acid	*N*-(2’-fluorocinnamoyl)-anthranilic acid	H	H	F	H	H	H	C_16_H_12_FNO_3_	284.0728	284.0734	-2.11	13.51	6
3-fluorocinnamic acid	anthranilic acid	*N*-(3’-fluorocinnamoyl)-anthranilic acid	H	H	H	F	H	H	C_16_H_12_FNO_3_	284.0728	284.0734	-2.11	13.49	7
4-fluorocinnamic acid	anthranilic acid	*N*-(4’-fluorocinnamoyl)-anthranilic acid	H	H	H	H	F	H	C_16_H_12_FNO_3_	284.0728	284.0722	2.11	13.45	8
2-chlorocinnamic acid	anthranilic acid	*N*-(2’-chlorocinnamoyl)-anthranilic acid	H	H	Cl	H	H	H	C_16_H_12_ClNO_3_	300.0433	300.0433	0.00	13.99	9
2-trifluoromethylcinnamic acid	anthranilic acid	*N*-(2’-trifluoromethylcinnamoyl)-anthranilic acid	H	H	CF_3_	H	H	H	C_17_H_12_F_3_NO_3_	334.0697	334.0713	-4.79	14.08	10
3-trifluoromethylcinnamic acid	anthranilic acid	*N*-(3’-trifluoromethylcinnamoyl)-anthranilic acid	H	H	H	CF_3_	H	H	C_17_H_12_F_3_NO_3_	334.0697	334.0697	0.00	14.14	11
2-bromocinnamic acid	anthranilic acid	*N*-(2’-bromocinnamoyl)-anthranilic acid	H	H	Br	H	H	H	C_16_H_12_BrNO_3_	343.9928	343.9936	-2.32	14.16	12
3-bromocinnamic acid	anthranilic acid	*N*-(3’-bromocinnamoyl)-anthranilic acid	H	H	H	Br	H	H	C_16_H_12_BrNO_3_	343.9928	343.9939	-3.20	14.22	13
3-difluoromethoxycinnamic acid	anthranilic acid	*N*-(3’-difluoromethoxycinnamoyl)-anthranilic acid	H	H	H	OCHF_2_	H	H	C_17_H_13_F_2_NO_4_	332.0740	332.0741	-0.30	13.79	14
3-trifluoromethoxycinnamic acid	anthranilic acid	*N*-(3’-trifluoromethoxycinnamoyl)-anthranilic acid	H	H	H	OCF_3_	H	H	C_17_H_12_F_3_NO_4_	350.0646	350.0638	2.28	14.28	15
2-fluorocinnamic acid	3-hydroxyanthranilic acid	*N*-(2’-fluorocinnamoyl)-3-hydroxyanthranilic acid	H	OH	F	H	H	H	C_16_H_12_FNO_4_	300.0678	300.0679	-0.33	13.08	16
3-fluorocinnamic acid	3-hydroxyanthranilic acid	*N*-(3’-fluorocinnamoyl)-3-hydroxyanthranilic acid	H	OH	H	F	H	H	C_16_H_12_FNO_4_	300.0678	300.0686	-2.67	13.10	17
4-fluorocinnamic acid	3-hydroxyanthranilic acid	*N*-(4’-fluorocinnamoyl)-3-hydroxyanthranilic acid	H	OH	H	H	F	H	C_16_H_12_FNO_4_	300.0678	300.0663	4.99	13.08	18
2-chlorocinnamic acid	3-hydroxyanthranilic acid	*N*-(2’-chlorocinnamoyl)-3-hydroxyanthranilic acid	H	OH	Cl	H	H	H	C_16_H_12_ClNO_4_	316.0382	316.0386	-1.26	13.59	19
2-trifluoromethylcinnamic acid	3-hydroxyanthranilic acid	*N*-(2’-trifluoromethylcinnamoyl)-3-hydroxyanthranilic acid	H	OH	CF_3_	H	H	H	C_17_H_12_F_3_NO_4_	350.0646	350.0645	0.29	13.64	20
3-trifluoromethylcinnamic acid	3-hydroxyanthranilic acid	*N*-(3’-trifluoromethylcinnamoyl)-3-hydroxyanthranilic acid	H	OH	H	CF_3_	H	H	C_17_H_12_F_3_NO_4_	350.0646	350.0641	1.43	13.78	21
2-bromocinnamic acid	3-hydroxyanthranilic acid	*N*-(2’-bromocinnamoyl)-3-hydroxyanthranilic acid	H	OH	Br	H	H	H	C_16_H_12_BrNO_4_	359.9877	359.9885	-2.22	13.76	22
3-difluoromethoxycinnamic acid	3-hydroxyanthranilic acid	*N*-(3’-difluoromethoxycinnamoyl)-3-hydroxyanthranilic acid	H	OH	H	OCHF_2_	H	H	C_17_H_15_F_2_NO_5_	348.0689	348.0693	-1.15	13.45	23
3-trifluoromethoxycinnamic acid	3-hydroxyanthranilic acid	*N*-(3’-trifluoromethoxycinnamoyl)-3-hydroxyanthranilic acid	H	OH	H	OCF_3_	H	H	C_17_H_12_F_3_NO_5_	366.0595	366.0595	0.00	13.90	24
2-fluorocinnamic acid	3-methylanthranilic acid	*N*-(2’-fluorocinnamoyl)-3-methylanthranilic acid	H	CH_3_	F	H	H	H	C_17_H_14_FNO_3_	298.0885	298.0880	1.68	13.09	25
3-fluorocinnamic acid	3-methylanthranilic acid	*N*-(3’-fluorocinnamoyl)-3-methylanthranilic acid	H	CH_3_	H	F	H	H	C_17_H_14_FNO_3_	298.0885	298.0884	0.34	13.16	26
4-fluorocinnamic acid	3-methylanthranilic acid	*N*-(4’-fluorocinnamoyl)-3-methylanthranilic acid	H	CH_3_	H	H	F	H	C_17_H_14_FNO_3_	298.0885	298.0888	-1.00	13.10	27
2-chlorocinnamic acid	3-methylanthranilic acid	*N*-(2’-chlorocinnamoyl)-3-methylanthranilic acid	H	CH_3_	Cl	H	H	H	C_17_H_14_ClNO_3_	314.0589	314.0585	1.27	13.55	28
2-trifluoromethylcinnamic acid	3-methylanthranilic acid	*N*-(2’-trifluoromethylcinnamoyl)-3-methylanthranilic acid	H	CH_3_	CF_3_	H	H	H	C_18_H_14_F_3_NO_3_	348.0853	348.0853	0.00	13.81	29
3-trifluoromethylcinnamic acid	3-methylanthranilic acid	*N*-(3’-trifluoromethylcinnamoyl)-3-methylanthranilic acid	H	CH_3_	H	CF_3_	H	H	C_18_H_14_F_3_NO_3_	348.0853	348.0852	0.29	14.03	30
2-bromocinnamic acid	3-methylanthranilic acid	*N*-(2’-bromocinnamoyl)-3-methylanthranilic acid	H	CH_3_	Br	H	H	H	C_17_H_14_ClNO_3_	358.0084	358.0098	-3.91	13.71	31
3-bromocinnamic acid	3-methylanthranilic acid	*N*-(3’-bromocinnamoyl)-3-methylanthranilic acid	H	CH_3_	H	Br	H	H	C_17_H_14_ClNO_3_	358.0084	358.0091	-1.96	13.90	32
3-difluoromethoxycinnamic acid	3-methylanthranilic acid	*N*-(3’-difluoromethoxycinnamoyl)-3-methylanthranilic acid	H	CH_3_	H	OCHF_2_	H	H	C_18_H_15_F_2_NO_4_	346.0896	346.0893	0.87	13.60	33
3-trifluoromethoxycinnamic acid	3-methylanthranilic acid	*N*-(3’-trifluoromethoxycinnamoyl)-3-methylanthranilic acid	H	CH_3_	H	OCF_3_	H	H	C_18_H_14_F_3_NO_4_	364.0802	364.0801	0.27	14.18	34
4-fluorocinnamic acid	5-methylanthranilic acid	*N*-(4’-fluorocinnamoyl)-5-methylanthranilic acid	CH_3_	H	H	H	F	H	C_17_H_14_FNO_3_	298.0885	298.0871	4.70	11.70	35
4-bromocinnamic acid	5-methylanthranilic acid	*N*-(4’-bromocinnamoyl)-5-methylanthranilic acid	CH_3_	H	H	H	Br	H	C_17_H_14_BrNO_3_	358.0084	358.0073	3.07	12.86	36
2-fluorocinnamic acid	5-fluoroanthranilic acid	*N*-(2’-fluorocinnamoyl)-5-fluoroanthranilic acid	F	H	F	H	H	H	C_16_H_11_F_2_NO_3_	302.0634	302.0648	-4.64	13.07	37
2-chlorocinnamic acid	5-fluoroanthranilic acid	*N*-(2’-chlorocinnamoyl)-5-fluoroanthranilic acid	F	H	Cl	H	H	H	C_16_H_11_ClFNO_3_	318.0339	318.0334	1.57	13.58	38
3-difluoromethoxycinnamic acid	5-fluoroanthranilic acid	*N*-(3’-difluoromethoxycinnamoyl)-5-fluoroanthranilic acid	F	H	H	OCHF_2_	H	H	C_17_H_12_F_3_NO_4_	350.0646	350.0639	1.99	13.39	39
3-trifluoromethylcinnamic acid	5-fluoroanthranilic acid	*N*-(3’-trifluoromethylcinnamoyl)-5-fluoroanthranilic acid	F	H	H	CF_3_	H	H	C_17_H_11_F_4_NO_3_	352.0602	352.0601	0.28	13.74	40
2-chlorocinnamic acid	3-methoxyanthranilic acid	*N*-(2’-chlorocinnamoyl)-3-methoxyanthranilic acid	H	OCH_3_	Cl	H	H	H	C_17_H_14_ClNO_4_	330.0539	330.0525	3.99	13.02	41
2-bromocinnamic acid	3-methoxyanthranilic acid	*N*-(2’-bromocinnamoyl)-3-methoxyanthranilic acid	H	OCH_3_	Br	H	H	H	C_17_H_14_BrNO_4_	374.0033	374.0045	-3.21	13.17	42
2-fluorocinnamic acid	3-chloroanthranilic acid	*N*-(2’-fluorocinnamoyl)-3-chloroanthranilic acid	H	Cl	F	H	H	H	C_16_H_11_ClFNO_3_	318.0339	318.0344	-1.57	12.77	43
3-fluorocinnamic acid	3-chloroanthranilic acid	*N*-(3’-fluorocinnamoyl)-3-chloroanthranilic acid	H	Cl	H	F	H	H	C_16_H_11_ClFNO_3_	318.0339	318.0333	1.89	12.83	44
2-chlorocinnamic acid	3-chloroanthranilic acid	*N*-(2’-chlorocinnamoyl)-3-chloroanthranilic acid	H	Cl	Cl	H	H	H	C_16_H_11_Cl_2_NO_3_	334.0043	334.0034	2.69	13.18	45
3-difluoromethoxycinnamic acid	3-chloroanthranilic acid	*N*-(3’-difluoromethoxycinnamoyl)-3-chloroanthranilic acid	H	Cl	H	OCHF_2_	H	H	C_17_H_12_ClF_2_NO_4_	366.0350	366.0353	-0.82	13.29	46
2-trifluoromethylcinnamic acid	3-chloroanthranilic acid	*N*-(2’-trifluoromethylcinnamoyl)-3-chloroanthranilic acid	H	Cl	CF_3_	H	H	H	C_17_H_11_ClF_3_NO_3_	368.0307	368.0325	-4.89	13.40	47
3-trifluoromethylcinnamic acid	3-chloroanthranilic acid	*N*-(3’-trifluoromethylcinnamoyl)-3-chloroanthranilic acid	H	Cl	H	CF_3_	H	H	C_17_H_11_ClF_3_NO_3_	368.0307	368.0306	0.27	13.59	48
2-bromocinnamic acid	3-chloroanthranilic acid	*N*-(2’-bromocinnamoyl)-3-chloroanthranilic acid	H	Cl	Br	H	H	H	C_16_H_11_BrClNO_3_	377.9538	377.9520	4.76	13.33	49
3-trifluoromethoxycinnamic acid	3-chloroanthranilic acid	*N*-(3’-trifluoromethoxycinnamoyl)-3-chloroanthranilic acid	H	Cl	H	OCF_3_	H	H	C_17_H_11_ClF_3_NO_4_	384.0256	384.0251	1.30	13.74	50

^a^Mass accuracy = [(theoretical mass—measured mass) / (theoretical mass)] x 1.10^6^

### Production of dihydrocinnamoyl anthranilates

**Fig 3:**
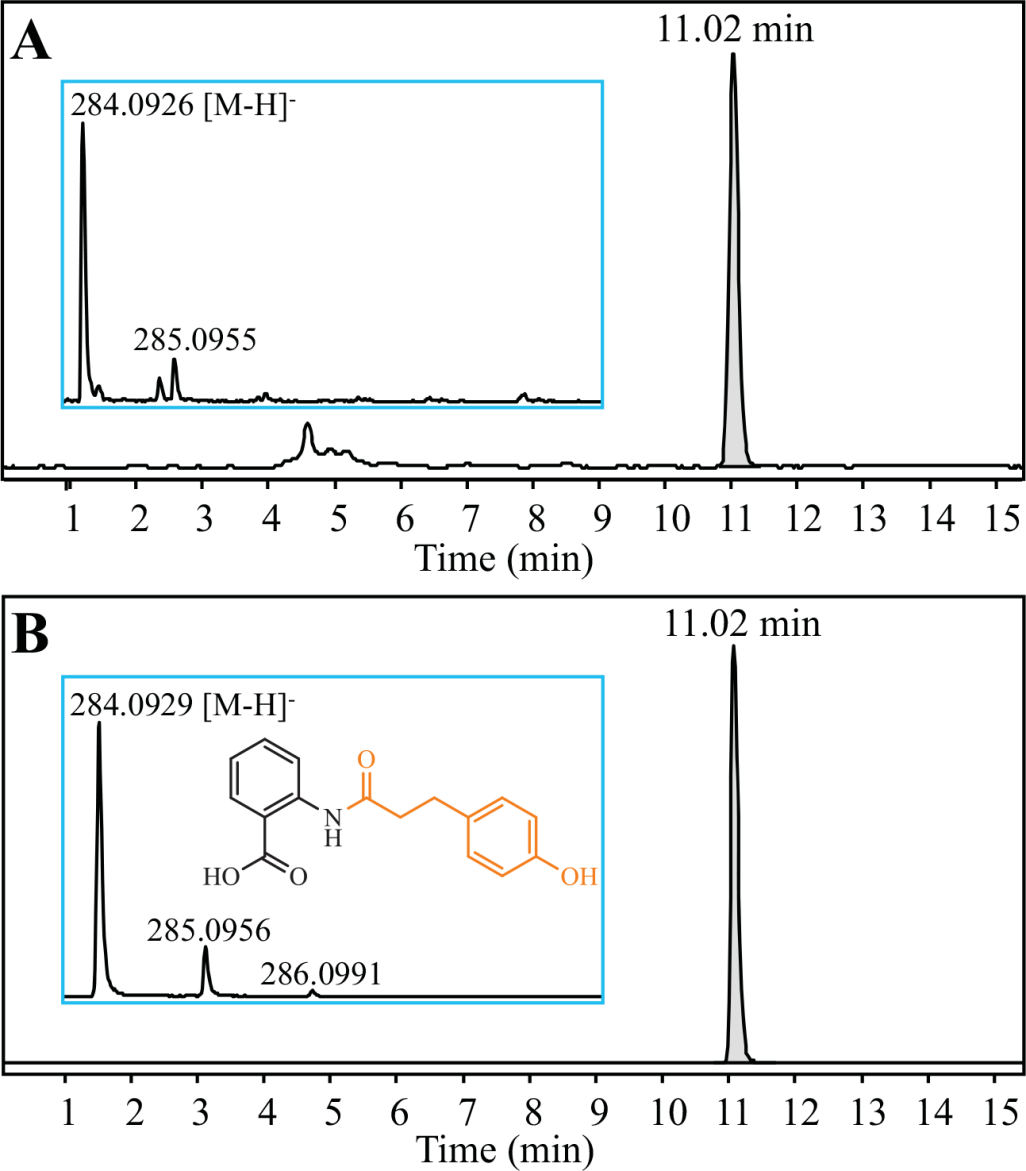
Detection of *N*-(4’-hydroxydihydrocinnamoyl)-anthranilate (DHavnD) from the recombinant yeast culture medium. Representative ESI-MS spectra were obtained after LC-TOF MS analysis of **(A)** the culture medium of recombinant yeast incubated with anthranilate and 4-hydroxydihydrocinnamate, and **(B)** a DHavnD standard solution.

We attempted to produce dihydrocinnamoyl anthranilates by feeding the yeast strain with various dihydrocinnamates (i.e., 3-phenylpropionate derivatives) and anthranilates. First, by comparison with the LC-MS elution profile of an authentic standard, the production of DHavnD (4.03 ± 0.08 μM) was successfully achieved by feeding 4-hydroxydihydrocinnamate and anthranilate (Fig 3), which indicated the promiscuity of 4CL5 and HCBT to use as substrates the saturated propanoid tail of cinnamate and cinnamoyl-CoA, respectively. No DHavnD was detected from the culture medium of control strains, fed with the same precursors and expressing either 4CL5 or HCBT alone. Next, as a preliminary round of screening, the medium of the engineered yeast was supplied with a series of 22 dihydrocinnamates (including halogenated dihydrocinnamates) in combination with anthranilate, which led to the production of 14 individual dihydrocinnamoyl anthranilates, according to the LC-MS analysis of the medium (Table 2, S2 Fig). The dihydrocinnamates that yielded a detectable product in the first round of screening were then co-fed with 3-hydroxyanthranilate or 3-methylanthranilate, which resulted in the production of 13 additional dihydrocinnamoyl anthranilates (Table 2, S2 Fig). The new compounds identified were not produced in the control yeast cultures fed only with anthranilates, demonstrating again the substrate promiscuity of both 4CL5 and HCBT enzymes in our in vivo production system.

**Table 2:** Structural characteristics of the dihydrocinnamoyl anthranilates (general structure shown in Fig 1B) produced in yeast and their identification based on dominant ion masses in ESI-MS spectra. Values were obtained from single feeding experiments for each combination of precursors.

Donor	Acceptor	Hydrogenated cinnamoyl anthranilates	R_1_	R_2_	R_3_	R_4_	R_5_	R_6_	Formula	Theoretical mass [M-H]^-^	Measured mass [M-H]^-^	Mass accuracy^a^ (ppm)	Retention time (min)(min)	Mass spectrum # in S2 Fig.
4-hydroxydihydrocinnamic acid	anthranilic acid	*N*-(4-hydroxydihydrocinnamoyl)-anthranilic acid (DHavnD)	H	H	H	H	OH	H	C_16_H_15_NO_4_	284.0928	284.0926	0.70	11.02	(Fig 3)
dihydrocinnamic acid	anthranilic acid	*N*-(dihydrocinnamoyl)-anthranilic acid	H	H	H	H	H	H	C_16_H_15_NO_3_	268.0979	268.0977	0.74	13.21	1
3-methyldihydrocinnamic acid	anthranilic acid	*N*-(3’-methyldihydrocinnamoyl)-anthranilic acid	H	H	H	CH_3_	H	H	C_17_H_17_NO_3_	282.1136	282.1136	0.00	13.65	2
4-methyldihydrocinnamic acid	anthranilic acid	*N*-(4’-methyldihydrocinnamoyl)-anthranilic acid	H	H	H	H	CH_3_	H	C_17_H_17_NO_3_	282.1136	282.1135	0.35	13.65	3
2-hydroxydihydrocinnamic acid	anthranilic acid	*N*-(2’-hydroxydihydrocinnamoyl)-anthranilic acid	H	H	OH	H	H	H	C_16_H_15_NO_4_	284.0928	284.0932	-1.40	12.22	4
3-hydroxydihydrocinnamic acid	anthranilic acid	*N*-(3’-hydroxydihydrocinnamoyl)-anthranilic acid	H	H	H	OH	H	H	C_16_H_15_NO_4_	284.0928	284.0934	-2.11	11.30	5
3-fluorodihydrocinnamic acid	anthranilic acid	*N*-(3’-fluorodihydrocinnamoyl)-anthranilic acid	H	H	H	F	H	H	C_16_H_14_FNO_3_	286.0885	286.0884	0.35	13.36	6
4-fluorodihydrocinnamic acid	anthranilic acid	*N*-(4’-fluorodihydrocinnamoyl)-anthranilic acid	H	H	H	H	F	H	C_16_H_14_FNO_3_	286.0885	286.0880	1.75	13.33	7
2-methoxydihydrocinnamic acid	anthranilic acid	*N*-(2’-methoxydihydrocinnamoyl)-anthranilic acid	H	H	OCH_3_	H	H	H	C_17_H_17_NO_4_	298.1084	298.1082	0.67	13.51	8
3-methoxydihydrocinnamic acid	anthranilic acid	*N*-(3’-methoxydihydrocinnamoyl)-anthranilic acid	H	H	H	OCH_3_	H	H	C_17_H_17_NO_4_	298.1084	298.1080	1.34	13.18	9
3,4-dihydroxydihydrocinnamic acid	anthranilic acid	*N*-(3’,4’-dihydroxydihydrocinnamoyl)-anthranilic acid	H	H	H	OH	OH	H	C_16_H_15_NO_5_	300.0877	300.0872	1.67	9.79	10
3-chlorodihydrocinnamic acid	anthranilic acid	*N*-(3’-chlorodihydrocinnamoyl)-anthranilic acid	H	H	H	Cl	H	H	C_16_H_14_ClNO_3_	302.0589	302.0581	2.65	13.91	11
3-methoxy-4-hydroxydihydrocinnamic acid	anthranilic acid	*N*-(3’-methoxy-4’-hydroxydihydrocinnamoyl)-anthranilic acid	H	H	H	OCH_3_	OH	H	C_17_H_17_NO_5_	314.1034	314.1032	0.64	11.30	12
2,5-dimethoxydihydrocinnamic acid	anthranilic acid	*N*-(2’,5’-dimethoxydihydrocinnamoyl)-anthranilic acid	H	H	OCH_3_	H	H	OCH_3_	C_18_H_19_NO_5_	328.1190	328.1201	-3.35	13.34	13
3,5-dimethoxy-4-hydroxydihydrocinnamic acid	anthranilic acid	*N*-(3’,5’-dimethoxy-4’-hydroxydihydrocinnamoyl)-anthranilic acid	H	H	H	OCH_3_	OH	OCH_3_	C_18_H_19_NO6	344.1140	344.1147	2.03	11.03	14
dihydrocinnamic acid	3-hydroxyanthranilic acid	*N*-(dihydrocinnamoyl)-3-hydroxyanthranilic acid	H	OH	H	H	H	H	C_16_H_15_NO_4_	284.0928	284.0931	-1.06	12.71	15
4-methyldihydrocinnamic acid	3-hydroxyanthranilic acid	*N*-(4’-methyldihydrocinnamoyl)-3-hydroxyanthranilic acid	H	OH	H	H	CH_3_	H	C_17_H_17_NO_4_	298.1085	298.1079	2.01	13.24	16
3-hydroxydihydrocinnamic acid	3-hydroxyanthranilic acid	*N*-(3’-hydroxydihydrocinnamoyl)-3-hydroxyanthranilic acid	H	OH	H	OH	H	H	C_16_H_15_NO_5_	300.0877	300.0881	-1.33	10.48	17
4-hydroxydihydrocinnamic acid	3-hydroxyanthranilic acid	*N*-(4’-hydroxydihydrocinnamoyl)-3-hydroxyanthranilic acid	H	OH	H	H	OH	H	C_16_H_15_NO_5_	300.0877	300.0878	-0.33	10.22	18
3-fluorodihydrocinnamic acid	3-hydroxyanthranilic acid	*N*-(3’-fluorodihydrocinnamoyl)-3-hydroxyanthranilic acid	H	OH	H	F	H	H	C_16_H_14_FNO_4_	302.0834	302.0837	-0.99	12.86	19
2-methoxydihydrocinnamic acid	3-hydroxyanthranilic acid	*N*-(2’-methoxydihydrocinnamoyl)-3-hydroxyanthranilic acid	H	OH	OCH_3_	H	H	H	C_17_H_17_NO_5_	314.1034	314.1027	2.23	13.06	20
3-methoxydihydrocinnamic acid	3-hydroxyanthranilic acid	*N*-(3’-methoxydihydrocinnamoyl)-3-hydroxyanthranilic acid	H	OH	H	OCH_3_	H	H	C_17_H_17_NO_5_	314.1034	314.1022	3.82	12.73	21
3-methoxy-4-hydroxydihydrocinnamic acid	3-hydroxyanthranilic acid	*N*-(3’-methoxy-4’-hydroxydihydrocinnamoyl)-3-hydroxyanthranilic acid	H	OH	H	OCH_3_	OH	H	C_17_H_17_NO_6_	330.0983	330.0980	0.91	10.52	22
2,5-dimethoxydihydrocinnamic acid	3-hydroxyanthranilic acid	*N*-(2’,5’-dimethoxydihydrocinnamoyl)-3-hydroxyanthranilic acid	H	OH	OCH_3_	H	OH	OCH_3_	C_18_H_19_NO_6_	344.1140	344.1139	0.29	12.92	23
4-hydroxydihydrocinnamic acid	3-methylanthranilic acid	*N*-(4’-hydroxydihydrocinnamoyl)-3-methylyanthranilic acid	H	CH_3_	H	H	OH	H	C_17_H_17_NO_4_	298.1085	298.1074	3.69	10.09	24
3,4-dihydroxydihydrocinnamic acid	3-methylanthranilic acid	*N*-(3’,4’-dihydroxydihydrocinnamoyl)-3-methylanthranilic acid	H	CH_3_	H	OH	OH	H	C_17_H_17_NO_5_	314.1034	314.1034	0.00	8.91	25
3-methoxy-4-hydroxydihydrocinnamic acid	3-methylanthranilic acid	*N*-(3’-methoxy-4’-hydroxydihydrocinnamoyl)-3-methylanthranilic acid	H	CH_3_	H	OCH_3_	OH	H	C_18_H_19_NO_5_	328.1190	328.1198	-2.44	10.39	26
2,5-dimethoxydihydrocinnamic acid	3-methylanthranilic acid	*N*-(2’,5’-dimethoxydihydrocinnamoyl)-3-methylanthranilic acid	H	CH_3_	OCH_3_	H	OH	OCH_3_	C_19_H_21_NO_5_	342.1347	342.1337	2.92	12.98	27

^a^Mass accuracy = [(theoretical mass—measured mass) / (theoretical mass)] x 1.10^6^

### Production of benzoyl anthranilates

**Fig 4:**
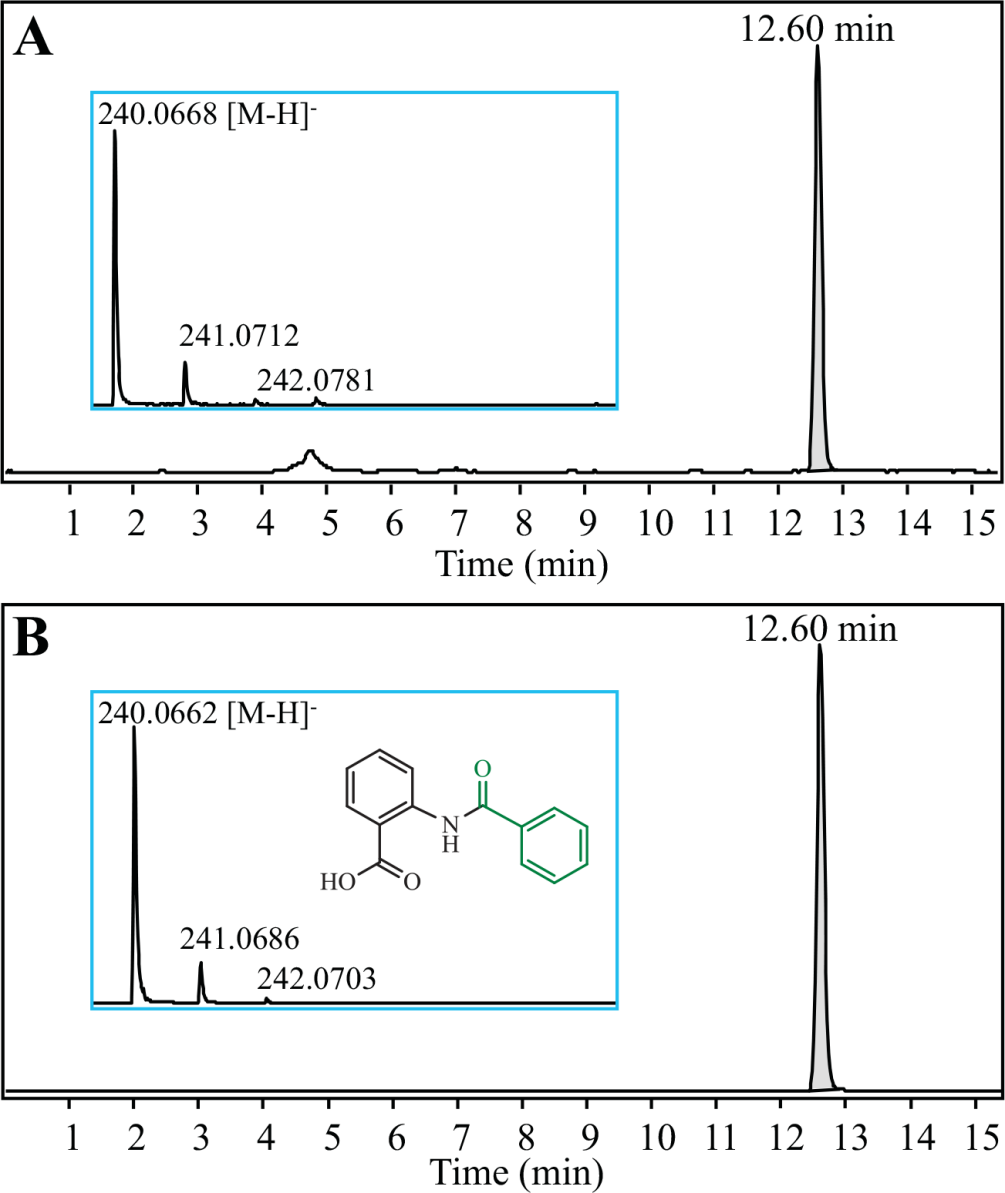
Detection of *N*-(benzoyl)-anthranilate (dianthramide B) from the recombinant yeast culture medium. Representative ESI-MS spectra were obtained after LC-TOF MS analysis of **(A)** the culture medium of recombinant yeast incubated with anthranilate and benzoic acid, and **(B)** a dianthramide B standard solution.

The production of benzoyl anthranilates by the 4CL5-HCBT yeast strain was tested because of the capacity of HCBT to use benzoyl-CoA as a donor in addition to coumaroyl-CoA [30]. We first successfully produced a benzoyl anthranilate named dianthramide B (1.20 ± 0.12 μM), by feeding the 4CL5- and HCBT-expressing yeast strain with benzoic acid and anthranilate. The identity of this new compound, which was detected directly from the culture medium, was confirmed with the authentic standard that exhibits the same LC-MS elution profile and mass (Fig 4), and by its absence in control cultures of strains expressing either 4CL5 or HCBT alone. Considering this unexpected substrate affinity of 4CL5 for benzoic acid, we fed 75 benzoate derivatives in combination with anthranilate for the synthesis of the corresponding benzoyl conjugates. This preliminary screening resulted in the production of 34 individual benzoyl anthranilates, including halogenated benzyol anthranilates, which were detected directly from the culture medium by LC-MS analysis (Table 3, S3 Fig). A second round of production using 3-hydroxyanthranilate or 3-methylanthranilate instead of anthranilate in the culture medium led to the production of 50 additional benzoyl anthranilates (Table 3, S3 Fig), which were absent from the culture medium of the yeast strain fed only with the anthranilates. These results demonstrate the capacity for 4CL5 to ligate coenzyme A onto at least 34 benzoate analogs; and the capacity for HCBT to conjugate the corresponding benzoyl-CoAs with various anthranilates.

**Table 3:** Structural characteristics of the benzoyl anthranilates (general structure shown in Fig 1C) produced in yeast and their identification based on dominant ion masses in ESI-MS spectra. Values were obtained from single feeding experiments for each combination of precursors.

Donor	Acceptor	Benzoyl anthranilates	R_1_	R_2_	R_3_	R_4_	R_5_	R_6_	Formula	Theoretical mass [M-H]^-^	Measured mass [M-H]^-^	Mass accuracy^a^ (ppm)	Retention time (min)	Mass spectrum # in S3 Fig.
benzoic acid	anthranilic acid	*N*-(benzoyl)-anthranilic acid (dianthramide B)	H	H	H	H	H	H	C_14_H_11_NO_3_	240.0666	240.0668	-0.83	12.60	(Fig 4)
3-aminobenzoic acid	anthranilic acid	*N*-(3’-aminobenzoyl)-anthranilic acid	H	H	H	NH_2_	H	H	C_14_H_12_NO_3_	255.0775	255.0774	0.39	10.39	1
2-methylbenzoic acid	anthranilic acid	*N*-(2’-methylbenzoyl)-anthranilic acid	H	H	CH_3_	H	H	H	C_15_H_13_NO_3_	254.0823	254.0823	0.00	13.19	2
3-methylbenzoic acid	anthranilic acid	*N*-(3’-methylbenzoyl)-anthranilic acid	H	H	H	CH_3_	H	H	C_15_H_13_NO_3_	254.0822	254.0825	-1.18	13.18	3
4-methylbenzoic acid	anthranilic acid	*N*-(4’-methylbenzoyl)-anthranilic acid	H	H	H	H	CH_3_	H	C_15_H_13_NO_3_	254.0822	254.0825	-1.18	13.16	4
3-hydroxybenzoic acid	anthranilic acid	*N*-(3’-hydroxybenzoyl)-anthranilic acid	H	H	H	OH	H	H	C_14_H_11_NO_4_	256.0615	256.0628	-2.73	10.68	5
4-hydroxybenzoic acid	anthranilic acid	*N*-(4’-hydroxybenzoyl)-anthranilic acid	H	H	H	H	OH	H	C_14_H_11_NO_4_	256.0615	256.0610	1.95	10.62	6
2-fluorobenzoic acid	anthranilic acid	*N*-(2’-fluorobenzoyl)-anthranilic acid	H	H	F	H	H	H	C_14_H_10_FNO_3_	258.0572	258.0577	-1.68	12.73	7
3-fluorobenzoic acid	anthranilic acid	*N*-(3’-fluorobenzoyl)-anthranilic acid	H	H	H	F	H	H	C_14_H_10_FNO_3_	258.0572	258.0566	2.32	12.90	8
4-fluorobenzoic acid	anthranilic acid	*N*-(4’-fluorobenzoyl)-anthranilic acid	H	H	H	H	F	H	C_14_H_10_FNO_3_	258.0572	258.0573	-0.39	12.83	9
2,5-dimethylbenzoic acid	anthranilic acid	*N*-(2’,5’-dimethylbenzoyl)-anthranilic acid	H	H	CH_3_	H	H	CH_3_	C_16_H_15_NO_3_	268.0979	268.0982	-1.12	13.58	10
3,4-dimethylbenzoic acid	anthranilic acid	*N*-(3’,4’-dimethylbenzoyl)-anthranilic acid	H	H	H	CH_3_	CH_3_	H	C_16_H_15_NO_3_	268.0979	268.0981	-0.75	13.52	11
3,5-dimethylbenzoic acid	anthranilic acid	*N*-(3’,5’-dimethylbenzoyl)-anthranilic acid	H	H	H	CH_3_	H	CH_3_	C_16_H_15_NO_3_	268.0979	268.0977	0.75	13.61	12
3-methoxybenzoic acid	anthranilic acid	*N*-(3’-methoxybenzoyl)-anthranilic acid	H	H	H	OCH_3_	H	H	C_15_H_13_NO_4_	270.0772	270.0777	-1.85	12.86	13
4-methoxybenzoic acid	anthranilic acid	*N*-(4’-methoxybenzoyl)-anthranilic acid	H	H	H	H	OCH_3_	H	C_15_H_13_NO_4_	270.0772	270.0770	0.74	12.74	14
4-hydroxymethylbenzoic acid	anthranilic acid	*N*-(4’-hydroxymethylbenzoyl)-anthranilic acid	H	H	H	H	CH_2_OH	H	C_15_H_13_NO_4_	270.0772	270.0779	-2.59	9.92	15
2-amino-5-methylbenzoic acid	anthranilic acid	*N*-(2’-amino-5’-methylbenzoyl)-anthranilic acid	H	H	NH_2_	H	H	CH_3_	C_15_H_14_N_2_O_3_	269.0932	269.0936	-1.48	13.03	16
2-amino-3-hydroxybenzoic acid	anthranilic acid	*N*-(2’-amino-3’-hydroxybenzoyl)-anthranilic acid	H	H	NH_2_	OH	H	H	C_14_H_12_N_2_O_4_	271.0724	271.0713	4.05	13.06	17
2-chlorobenzoic acid	anthranilic acid	*N*-(2’-chlorobenzoyl)-anthranilic acid	H	H	Cl	H	H	H	C_14_H_10_ClNO_3_	274.02765	274.0279	-0.93	13.48	18
3-chlorobenzoic acid	anthranilic acid	*N*-(3’-chlorobenzoyl)-anthranilic acid	H	H	H	Cl	H	H	C_14_H_10_ClNO_3_	274.02765	274.0272	1.64	13.54	19
4-chlorobenzoic acid	anthranilic acid	*N*-(4’-chlorobenzoyl)-anthranilic acid	H	H	H	H	Cl	H	C_14_H_10_ClNO_3_	274.02764	274.0266	3.81	13.48	20
3-dimethylaminobenzoic acid	anthranilic acid	*N*-(3’-dimethylaminobenzoyl)-anthranilic acid	H	H	H	N(CH_3_)_2_	H	H	C_16_H_16_N_2_O_3_	283.1088	283.1081	2.47	13.25	21
4-dimethylaminobenzoic acid	anthranilic acid	*N*-(4’-dimethylaminobenzoyl)-anthranilic acid	H	H	H	H	N(CH_3_)_2_	H	C_16_H_16_N_2_O_3_	283.1088	283.1083	1.77	13.24	22
4-nitrobenzoic acid	anthranilic acid	*N*-(4’-nitrobenzoyl)-anthranilic acid	H	H	H	H	NO_2_	H	C_14_H_10_N_2_O_5_	285.0517	285.0519	-0.70	8.10	23
3-methoxy-4-hydroxybenzoic acid	anthranilic acid	*N*-(3’-methoxy-4’-hydroxybenzoyl)-anthranilic acid	H	H	H	OCH_3_	OH	H	C_15_H_13_NO_5_	286.0721	286.0725	-1.40	10.94	24
3-methylthiobenzoic acid	anthranilic acid	*N*-(3’-methylthiobenzoyl)-anthranilic acid	H	H	H	SCH_3_	H	H	C_15_H_13_NO_3_S	286.0543	286.0545	-0.56	13.60	25
4-methylthiobenzoic acid	anthranilic acid	*N*-(4’-methylthiobenzoyl)-anthranilic acid	H	H	H	H	SCH_3_	H	C_15_H_13_NO_3_S	286.0543	286.0547	-1.39	13.47	26
3,4-dimethoxybenzoic acid	anthranilic acid	*N*-(3’,4’-dimethoxybenzoyl)-anthranilic acid	H	H	H	OCH_3_	OCH_3_	H	C_16_H_15_NO5	300.0877	300.0863	4.66	13.15	27
3-trifluoromethylbenzoic acid	anthranilic acid	*N*-(3’-trifluoromethylbenzoyl)-anthranilic acid	H	H	H	CF_3_	H	H	C_15_H_10_F_3_NO_3_	308.0540	308.0546	-1.94	13.76	28
4-trifluoromethylbenzoic acid	anthranilic acid	*N*-(4’-trifluoromethylbenzoyl)-anthranilic acid	H	H	H	H	CF_3_	H	C_15_H_10_F_3_NO_3_	308.0540	308.0539	0.32	13.60	29
3-bromobenzoic acid	anthranilic acid	*N*-(3’-bromobenzoyl)-anthranilic acid	H	H	H	Br	H	H	C_14_H_10_BrNO_3_	317.9771	317.9777	-1.88	13.78	30
3-trifluoromethoxybenzoic acid	anthranilic acid	*N*-(3’-trifluoromethoxybenzoyl)-anthranilic acid	H	H	H	OCF_3_	H	H	C_15_H_10_F_3_NO_4_	324.0489	324.0495	1.85	13.95	31
4-trifluoromethoxybenzoic acid	anthranilic acid	*N*-(4’-trifluoromethoxybenzoyl)-anthranilic acid	H	H	H	H	OCF_3_	H	C_15_H_10_F_3_NO_4_	324.0489	324.0489	0.00	13.76	32
3-iodobenzoic acid	anthranilic acid	*N*-(3’-iodobenzoyl)-anthranilic acid	H	H	H	I	H	H	C_14_H_10_INO_3_	365.9633	365.9642	-2.46	14.03	33
4-iodobenzoic acid	anthranilic acid	*N*-(4’-iodobenzoyl)-anthranilic acid	H	H	H	H	I	H	C_14_H_10_INO_3_	365.9633	365.9633	0.00	13.91	34
benzoic acid	3-hydroxyanthranilic acid	*N*-(benzoyl)-3-hydroxyanthranilic acid	H	OH	H	H	H	H	C_14_H_11_NO_4_	256.0615	256.0622	-2.73	11.94	35
3-aminobenzoic acid	3-hydroxyanthranilic acid	*N*-(3’-aminobenzoyl)-3-hydroxyanthranilic acid	H	OH	H	NH_2_	H	H	C_14_H_12_N_2_O_4_	271.0724	271.0728	-1.47	9.90	36
3-methylbenzoic acid	3-hydroxyanthranilic acid	*N*-(3’-methylbenzoyl)-3-hydroxyanthranilic acid	H	OH	H	CH_3_	H	H	C_15_H_13_NO_4_	270.0772	270.0773	-0.37	12.78	37
4-methylbenzoic acid	3-hydroxyanthranilic acid	*N*-(4’-methylbenzoyl)-3-hydroxyanthranilic acid	H	OH	H	H	CH_3_	H	C_15_H_13_NO_4_	270.0772	270.0773	-0.37	12.75	38
3-hydroxybenzoic acid	3-hydroxyanthranilic acid	*N*-(3’-hydroxybenzoyl)-3-hydroxyanthranilic acid	H	OH	H	OH	H	H	C_14_H_11_NO_5_	272.0564	272.0575	-4.04	10.12	39
2-fluorobenzoic acid	3-hydroxyanthranilic acid	*N*-(2’-fluorobenzoyl)-3-hydroxyanthranilic acid	H	OH	F	H	H	H	C_14_H_10_FNO_4_	274.0521	274.0527	-2.19	11.70	40
3-fluorobenzoic acid	3-hydroxyanthranilic acid	*N*-(3’-fluorobenzoyl)-3-hydroxyanthranilic acid	H	OH	H	F	H	H	C_14_H_10_FNO_4_	274.0521	274.0522	-0.36	12.33	41
4-fluorobenzoic acid	3-hydroxyanthranilic acid	*N*-(4’-fluorobenzoyl)-3-hydroxyanthranilic acid	H	OH	H	H	F	H	C_14_H_10_FNO_4_	274.0521	274.0518	1.09	12.30	42
3,4-dimethylbenzoic acid	3-hydroxyanthranilic acid	*N*-(3’,4’-dimethylbenzoyl)-3-hydroxyanthranilic acid	H	OH	H	CH_3_	CH_3_	H	C_16_H_15_NO_4_	284.0928	284.0925	1.06	13.17	43
3,5-dimethylbenzoic acid	3-hydroxyanthranilic acid	*N*-(3’,5’-dimethylbenzoyl)-3-hydroxyanthranilic acid	H	OH	H	CH_3_	H	CH_3_	C_16_H_15_NO_4_	284.0928	284.0929	-0.35	13.27	44
3-methoxybenzoic acid	3-hydroxyanthranilic acid	*N*-(3’-methoxybenzoyl)-3-hydroxyanthranilic acid	H	OH	H	OCH_3_	H	H	C_15_H_13_NO_5_	286.0721	286.0723	-0.70	12.38	45
4-methoxybenzoic acid	3-hydroxyanthranilic acid	*N*-(4’-methoxybenzoyl)-3-hydroxyanthranilic acid	H	OH	H	H	OCH_3_	H	C_15_H_13_NO_5_	286.0721	286.0722	-0.35	12.37	46
4-hydroxymethylbenzoic acid	3-hydroxyanthranilic acid	*N*-(4’-hydroxymethylbenzoyl)-3-hydroxyanthranilic acid	H	OH	H	H	CH_2_OH	H	C_15_H_13_NO_5_	286.0721	286.0719	-0.70	9.23	47
2-chlorobenzoic acid	3-hydroxyanthranilic acid	*N*-(2’-chlorobenzoyl)-3-hydroxyanthranilic acid	H	OH	Cl	H	H	H	C_14_H_10_ClNO_4_	290.0226	290.0221	1.72	12.95	48
3-chlorobenzoic acid	3-hydroxyanthranilic acid	*N*-(3’-chlorobenzoyl)-3-hydroxyanthranilic acid	H	OH	H	Cl	H	H	C_14_H_10_ClNO_4_	290.0226	290.0225	0.34	12.99	49
4-chlorobenzoic acid	3-hydroxyanthranilic acid	*N*-(4’-chlorobenzoyl)-3-hydroxyanthranilic acid	H	OH	H	H	Cl	H	C_14_H_10_ClNO_4_	290.0226	290.0223	1.03	12.95	50
3-dimethylaminobenzoic acid	3-hydroxyanthranilic acid	*N*-(3’-dimethylaminobenzoyl)-3-hydroxyanthranilic acid	H	OH	H	N(CH_3_)_2_	H	H	C_16_H_16_N_2_O_4_	299.1037	299.1038	-0.33	12.88	51
3-methoxy-4-hydroxybenzoic acid	3-hydroxyanthranilic acid	*N*-(3’-methoxy-4’-hydroxybenzoyl)-3-hydroxyanthranilic acid	H	OH	H	OCH_3_	OH	H	C_15_H_13_NO_6_	302.0670	302.0672	-0.66	10.33	52
3-methylthiobenzoic acid	3-hydroxyanthranilic acid	*N*-(3’-methylthiobenzoyl)-3-hydroxyanthranilic acid	H	OH	H	SCH_3_	H	H	C_15_H_13_NO_4_S	302.0493	302.0491	0.66	13.10	53
4-methylthiobenzoic acid	3-hydroxyanthranilic acid	*N*-(4’-methylthiobenzoyl)-3-hydroxyanthranilic acid	H	OH	H	H	SCH_3_	H	C_15_H_13_NO_4_S	302.0493	302.0499	-1.98	13.04	54
3,4-dimethoxybenzoic acid	3-hydroxyanthranilic acid	*N*-(3’,4’-dimethoxybenzoyl)-3-hydroxyanthranilic acid	H	OH	H	OCH_3_	OCH_3_	H	C_16_H_15_NO_6_	316.0827	316.0825	0.63	11.52	55
3-trifluoromethylbenzoic acid	3-hydroxyanthranilic acid	*N*-(3’-trifluoromethylbenzoyl)-3-hydroxyanthranilic acid	H	OH	H	CF_3_	H	H	C_15_H_10_F_3_NO_4_	324.0489	324.0487	0.62	13.22	56
4-trifluoromethylbenzoic acid	3-hydroxyanthranilic acid	*N*-(4’-trifluoromethylbenzoyl)-3-hydroxyanthranilic acid	H	OH	H	H	CF_3_	H	C_15_H_10_F_3_NO_4_	324.0489	324.0487	0.62	13.13	57
3-bromobenzoic acid	3-hydroxyanthranilic acid	*N*-(3’-bromobenzoyl)-3-hydroxyanthranilic acid	H	OH	H	Br	H	H	C_14_H_10_BrNO_4_	333.9720	333.9723	-0.90	13.20	58
3-trifluoromethoxybenzoic acid	3-hydroxyanthranilic acid	*N*-(3’-trifluoromethoxybenzoyl)-3-hydroxyanthranilic acid	H	OH	H	OCF_3_	H	H	C_15_H_10_F_3_NO_5_	340.0438	340.0436	0.59	13.38	60
4-trifluoromethoxybenzoic acid	3-hydroxyanthranilic acid	*N*-(4’-trifluoromethoxybenzoyl)-3-hydroxyanthranilic acid	H	OH	H	H	OCF_3_	H	C_15_H_10_F_3_NO_5_	340.0438	340.0446	-2.35	13.32	61
3-iodobenzoic acid	3-hydroxyanthranilic acid	*N*-(3’-iodobenzoyl)-3-hydroxyanthranilic acid	H	OH	H	I	H	H	C_14_H_10_INO_4_	381.9582	381.9580	0.52	13.54	62
4-iodobenzoic acid	3-hydroxyanthranilic acid	*N*-(4’-iodobenzoyl)-3-hydroxyanthranilic acid	H	OH	H	H	I	H	C_14_H_10_INO_4_	381.9582	381.9583	-0.26	13.42	63
3-methylbenzoic acid	3-methylanthranilic acid	*N*-(3’-methylbenzoyl)-3-methylanthranilic acid	H	CH_3_	H	CH_3_	H	H	C_16_H_15_NO_3_	268.0979	268.0978	0.37	12.95	64
3,4-dimethylbenzoic acid	3-methylanthranilic acid	*N*-(3’,4’-dimethylbenzoyl)-3-methylanthranilic acid	H	CH_3_	H	CH_3_	CH_3_	H	C_17_H_17_NO_3_	282.1136	282.1137	-0.35	13.39	65
3,5-dimethylbenzoic acid	3-methylanthranilic acid	*N*-(3’,5’-dimethylbenzoyl)-3-methylanthranilic acid	H	CH_3_	H	CH_3_	H	CH_3_	C_17_H_17_NO_3_	282.1136	282.1135	0.35	13.53	66
3-methoxybenzoic acid	3-methylanthranilic acid	*N*-(3’-methoxybenzoyl)-3-methylanthranilic acid	H	CH_3_	H	OCH_3_	H	H	C_16_H_15_NO_4_	284.0928	284.0925	1.06	12.60	67
4-methoxybenzoic acid	3-methylanthranilic acid	*N*-(4’-methoxybenzoyl)-3-methylanthranilic acid	H	CH_3_	H	H	OCH_3_	H	C_16_H_15_NO_4_	284.0928	284.0927	0.35	12.47	68
4-hydroxymethylbenzoic acid	3-methylanthranilic acid	*N*-(4’-hydroxymethylbenzoyl)-3-methylanthranilic acid	H	CH_3_	H	H	CH_2_OH	H	C_16_H_15_NO_4_	284.0928	284.0920	2.82	9.41	69
3-chlorobenzoic acid	3-methylanthranilic acid	*N*-(3’-chlorobenzoyl)-3-methylanthranilic acid	H	CH_3_	H	Cl	H	H	C_15_H_12_ClNO_3_	288.0433	288.0422	3.82	13.26	70
4-chlorobenzoic acid	3-methylanthranilic acid	*N*-(4’-chlorobenzoyl)-3-methylanthranilic acid	H	CH_3_	H	H	Cl	H	C_15_H_12_ClNO_3_	288.0433	288.0420	4.51	13.19	71
3-dimethylaminobenzoic acid	3-methylanthranilic acid	*N*-(3’-dimethylaminobenzoyl)-3-methylanthranilic acid	H	CH_3_	H	N(CH_3_)_2_	H	H	C_17_H_18_N_2_O_3_	297.1245	297.1250	-1.68	12.97	72
3-methoxy-4-hydroxybenzoic acid	3-methylanthranilic acid	*N*-(3’-methoxy-4’-hydroxybenzoyl)-3-methylanthranilic acid	H	CH_3_	H	OCH_3_	OH	H	C_16_H_15_NO_5_	300.0877	300.0877	0.00	10.34	73
3-methylthiobenzoic acid	3-methylanthranilic acid	*N*-(3’-methylthiobenzoyl)-3-methylanthranilic acid	H	CH_3_	H	SCH_3_	H	H	C_16_H_15_NO_3_S	300.0700	300.0704	-1.33	13.30	74
4-methylthiobenzoic acid	3-methylanthranilic acid	*N*-(4’-methylthiobenzoyl)-3-methylanthranilic acid	H	CH_3_	H	H	SCH_3_	H	C_16_H_15_NO_3_S	300.0700	300.0706	-1.99	13.22	75
3,4-dimethoxybenzoic acid	3-methylanthranilic acid	*N*-(3’,4’-dimethoxybenzoyl)-3-methylanthranilic acid	H	CH_3_	H	OCH_3_	OCH_3_	H	C_17_H_17_NO_5_	314.1034	314.1032	0.64	11.67	76
3-trifluoromethylbenzoic acid	3-methylanthranilic acid	*N*-(3’-trifluoromethylbenzoyl)-3-methylanthranilic acid	H	CH_3_	H	CF_3_	H	H	C_16_H_12_F_3_NO_3_	322.0697	322.0720	-7.14	13.56	77
4-trifluoromethylbenzoic acid	3-methylanthranilic acid	*N*-(4’-trifluoromethylbenzoyl)-3-methylanthranilic acid	H	CH_3_	H	H	CF_3_	H	C_16_H_12_F_3_NO_3_	322.0697	322.0688	2.79	13.55	78
3-bromobenzoic acid	3-methylanthranilic acid	*N*-(3’-bromobenzoyl)-3-methylanthranilic acid	H	CH_3_	H	Br	H	H	C_15_H_12_BrNO_3_	331.9928	331.9930	-0.60	13.43	79
4-bromobenzoic acid	3-methylanthranilic acid	*N*-(4’-bromobenzoyl)-3-methylanthranilic acid	H	CH_3_	H	H	Br	H	C_15_H_12_BrNO_3_	331.9928	331.9924	1.20	13.37	80
3-trifluoromethoxybenzoic acid	3-methylanthranilic acid	*N*-(3’-trifluoromethoxybenzoyl)-3-methylanthranilic acid	H	CH_3_	H	OCF_3_	H	H	C_16_H_12_F_3_NO_4_	338.0646	338.0646	0.00	13.75	81
4-trifluoromethoxybenzoic acid	3-methylanthranilic acid	*N*-(4’-trifluoromethoxybenzoyl)-3-methylanthranilic acid	H	CH_3_	H	H	OCF_3_	H	C_16_H_12_F_3_NO_4_	338.0646	338.0637	2.66	13.70	82
3-iodobenzoic acid	3-methylanthranilic acid	*N*-(3’-iodobenzoyl)-3-methylanthranilic acid	H	CH_3_	H	I	H	H	C_15_H_12_INO_3_	379.9789	379.9803	-3.68	13.75	83
4-iodobenzoic acid	3-methylanthranilic acid	*N*-(4’-iodobenzoyl)-3-methylanthranilic acid	H	CH_3_	H	H	I	H	C_15_H_12_INO_3_	379.9789	379.9789	0.00	13.70	84

^a^Mass accuracy = [(theoretical mass—measured mass) / (theoretical mass)] x 1.10^6^

## Discussion

With an emphasis on the class of cinnamyol, dihydrocinnamoyl, and benzoyl anthranilates, we illustrate in this study the possibility of producing numerous chemically diverse molecules using biological synthesis rather than conventional chemical synthesis. Our data imply that the promiscuity of 4CL5 allows the catalytic conversion of a great diversity of dihydrocinnamates, benzoates, and various cinnamates into the corresponding acyl-CoA-thioesters. To our knowledge, this is the first description of a bona fide 4-coumaroyl:CoA ligase (EC 6.2.1.12) showing benzoyl:CoA (EC 6.2.1.25), 3-hydroxybenzoyl:CoA (EC 6.2.1.37), 4-hydroxybenzoyl:CoA (EC 6.2.1.27), and 4-chlorobenzoyl:CoA (EC 6.2.1.33) ligase activities. Our original attempts to co-express HCBT with known bacterial benzoyl:CoA ligases for the production of benzoyl anthranilates in yeast were unsuccessful, possibly due to the high pH optima (pH > 8.5) of these enzymes [36,37]. Nevertheless, using the 4CL5 enzyme, we demonstrate the feasibility of producing a substantial diversity of benzoyl-CoA thioesters and benzoate conjugate molecules in yeast. This discovery opens new possibilities for the heterologous combinatorial production of valuable benzoylated metabolites such as benzylbenzoates; benzophenones; the anticancer drug taxol; polyketides with antimicrobial activities (e.g., wailupemycin, enterocin, soraphen A); and unnatural polyketides using engineered benzoyl-CoA-dependent polyketide synthases [38]. Furthermore, heterologously synthesized benzoyl anthranilates can be used as scaffolds for the synthesis of related anti-adenoviral compounds and oncogene inhibitors [39,40].

We observed the activity of 4CL5 towards various dihydrocinnamates and non-natural halogenated cinnamates and exploited its catalytic property to biosynthesize libraries of non-natural and structurally diverse cinnamoyl and dihydrocinnamoyl anthranilates using HCBT. For example, the drug DHavnD was synthesized, and utilization of alternate precursors resulted in the rapid production of 27 additional DHavnD analogs. These results point towards the eventual design of more biologically active drugs through the addition of halogens. They also illustrate the advantage of biological synthesis to achieve bifunctionalization, as exemplified by several of our bi-halogenated compounds. Finally, through co-expression with the adequate synthases, the capacity of 4CL5 to activate dihydrocinnamates creates the potential for biomanufacture of valuable natural products, such as the antibacterial dihydrocinnamoyl forms of flavans and chalcones [41,42].

The HCBT enzyme used in this study belongs to the BAHD enzyme family, which contains multiple members that catalyze the transfer of cinnamoyl- and benzoyl-CoAs into a great diversity of distinct acceptors [43]. Although HCBT offers flexibility for a wide range of acyl-CoA donors, its affinity towards acceptors seems limited to anthranilates. Therefore, engineering yeast strains that co-express 4CL5 with various BAHD transferases would considerably expand the type and number of molecules that can be biosynthesized heterologously.

Ultimately, biosynthesis of particular cinnamoyl or benzoyl anthranilates from renewable and inexpensive carbon sources could be desirable for cost-effective manufacturing. For this purpose, we recently demonstrated a *de novo* pathway for the production of *p*-coumarate and two avenanthramides from glucose in *E*. *coli* [35]. In this pathway, additional expression of hydroxycinnamoyl-CoA double-bond reductase could be used for the synthesis of dihydrocinnamates [44], whereas benzoate biosynthesis can be achieved from the aromatic amino acid phenylalanine [45]. Finally, the recent discovery of halogenases from bacteria and fungi has already proven to be useful for *de novo* synthesis of halogenated bioactive metabolites in microorganisms [46,47].

As a conclusion, the use of two promiscuous enzymes, 4CL5 and HCBT, demonstrates the potential to develop a platform for the precursor-directed combinatorial biosynthesis of cinnamoyl, dihydrocinnamoyl, and benzoyl anthranilates. In this study and in our previous work [27], this system using a single engineered yeast strain supported the production of more than 180 target metabolites belonging to cinnamoyl, dihydrocinnamoyl, or benzoyl anthranilate families. Moreover, we believe that testing our system with more substituted cinnamates and benzoates could result in the production of several additional metabolites.

## Supporting Information

S1 Fig LC-MS elution profiles of 50 novel cinnamoyl anthranilates produced by the recombinant 4CL5-HCBT yeast strain. ESI-MS spectra were obtained after LC-TOF MS analysis of the culture medium of the yeast strain fed with the precursors indicated in Table 1.
(PPTX)

S2 Fig LC-MS elution profiles of 27 dihydrocinnamoyl anthranilates produced by the recombinant 4CL5-HCBT yeast strain. ESI-MS spectra were obtained after LC-TOF MS analysis of the culture medium of the yeast strain fed with the precursors indicated in Table 2.
(PPTX)

S3 Fig LC-MS elution profiles of 84 benzoyl anthranilates produced by the recombinant 4CL5-HCBT yeast strain. ESI-MS spectra were obtained after LC-TOF MS analysis of the culture medium of the yeast strain fed with the precursors indicated in Table 3.
(PPTX)

S1 Table Structures and concentrations of the cinnamates used for the yeast feedings. (DOCX)

S2 Table Structures and concentrations of the dihydrocinnamates used for the yeast feedings. (DOCX)

S3 Table Structures and concentrations of the benzoates used for the yeast feedings. (DOCX)
