# Supplementary material for: Precursor-Directed Combinatorial Biosynthesis of Cinnamoyl, Dihydrocinnamoyl, and Benzoyl Anthranilates in Saccharomyces cerevisiae
Source: PLoS One. 2015 Oct 2;10(10):e0138972. doi: 10.1371/journal.pone.0138972 (PMC4591981; doi:10.1371/journal.pone.0138972)
Supplement: S1 Fig — ESI-MS spectra were obtained after LC-TOF MS analysis of the culture medium of the yeast strain fed with the precursors indicated in Table 1. (PPTX) [file pone.0138972.s001.pptx]

## Slide 1
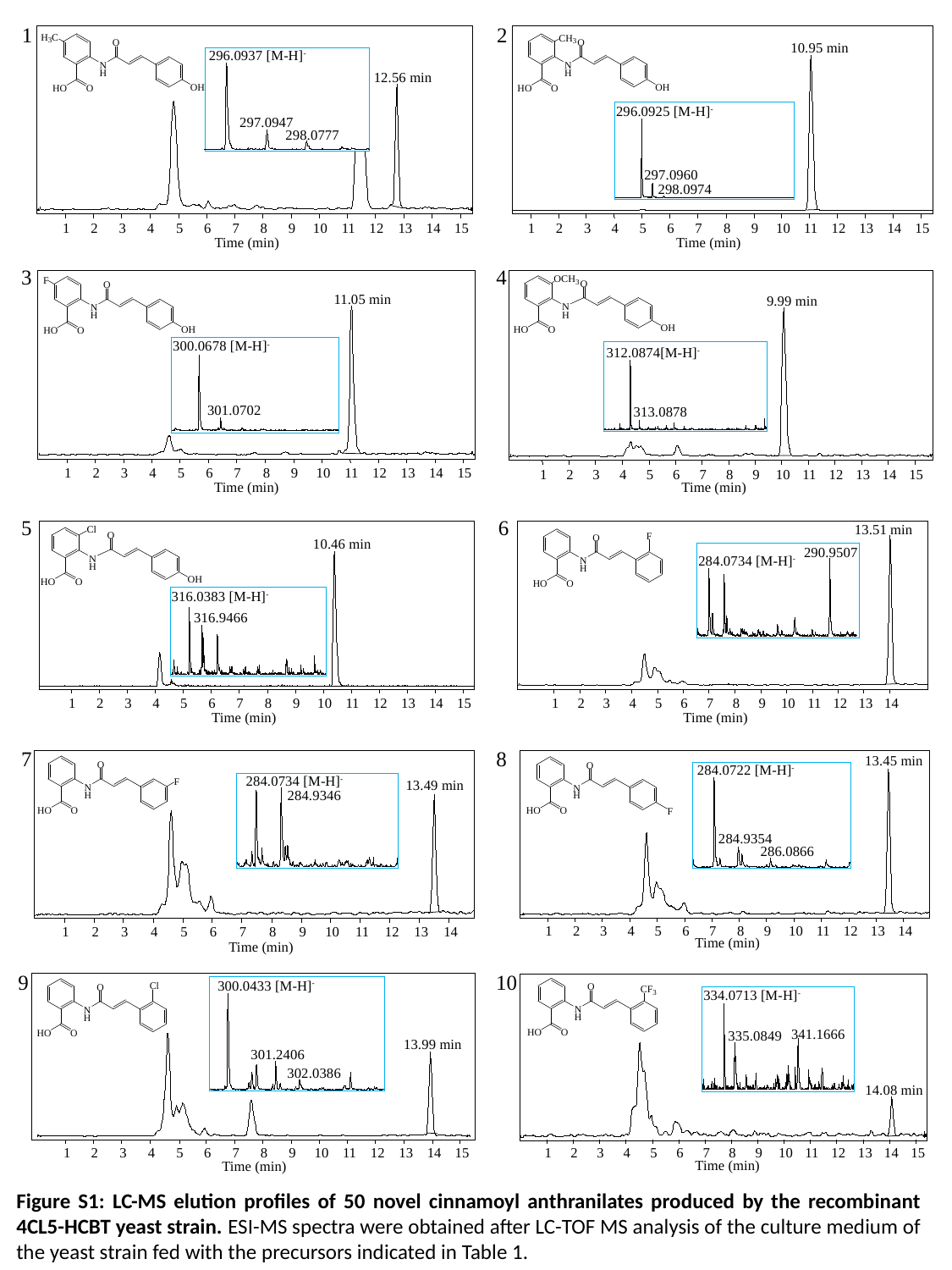

1
2
10.95 min
296.0937 [M-H]-
12.56 min
296.0925 [M-H]-
297.0947
298.0777
297.0960
298.0974
1
2
3
4
5
6
7
8
9
10
11
12
13
14
15
1
2
3
4
5
6
7
8
9
10
11
12
13
14
15
Time (min)
Time (min)
4
3
11.05 min
9.99 min
300.0678 [M-H]-
312.0874[M-H]-
301.0702
313.0878
1
2
3
4
5
6
7
8
9
10
11
12
13
14
15
1
2
3
4
5
6
7
8
9
10
11
12
13
14
15
Time (min)
Time (min)
5
6
13.51 min
10.46 min
290.9507
284.0734 [M-H]-
316.0383 [M-H]-
316.9466
1
2
3
4
5
6
7
8
9
10
11
12
13
14
1
2
3
4
5
6
7
8
9
10
11
12
13
14
15
Time (min)
Time (min)
8
7
284.0734 [M-H]-
13.49 min
284.9346
1
2
3
4
5
6
7
8
9
10
11
12
13
14
Time (min)
13.45 min
284.0722 [M-H]-
284.9354
286.0866
1
2
3
4
5
6
7
8
9
10
11
12
13
14
Time (min)
10
9
300.0433 [M-H]-
13.99 min
301.2406
302.0386
1
2
3
4
5
6
7
8
9
10
11
12
13
14
15
Time (min)
334.0713 [M-H]-
341.1666
335.0849
14.08 min
1
2
3
4
5
6
7
8
9
10
11
12
13
14
15
Time (min)
Figure S1: LC-MS elution profiles of 50 novel cinnamoyl anthranilates produced by the recombinant 4CL5-HCBT yeast strain. ESI-MS spectra were obtained after LC-TOF MS analysis of the culture medium of the yeast strain fed with the precursors indicated in Table 1.

## Slide 2
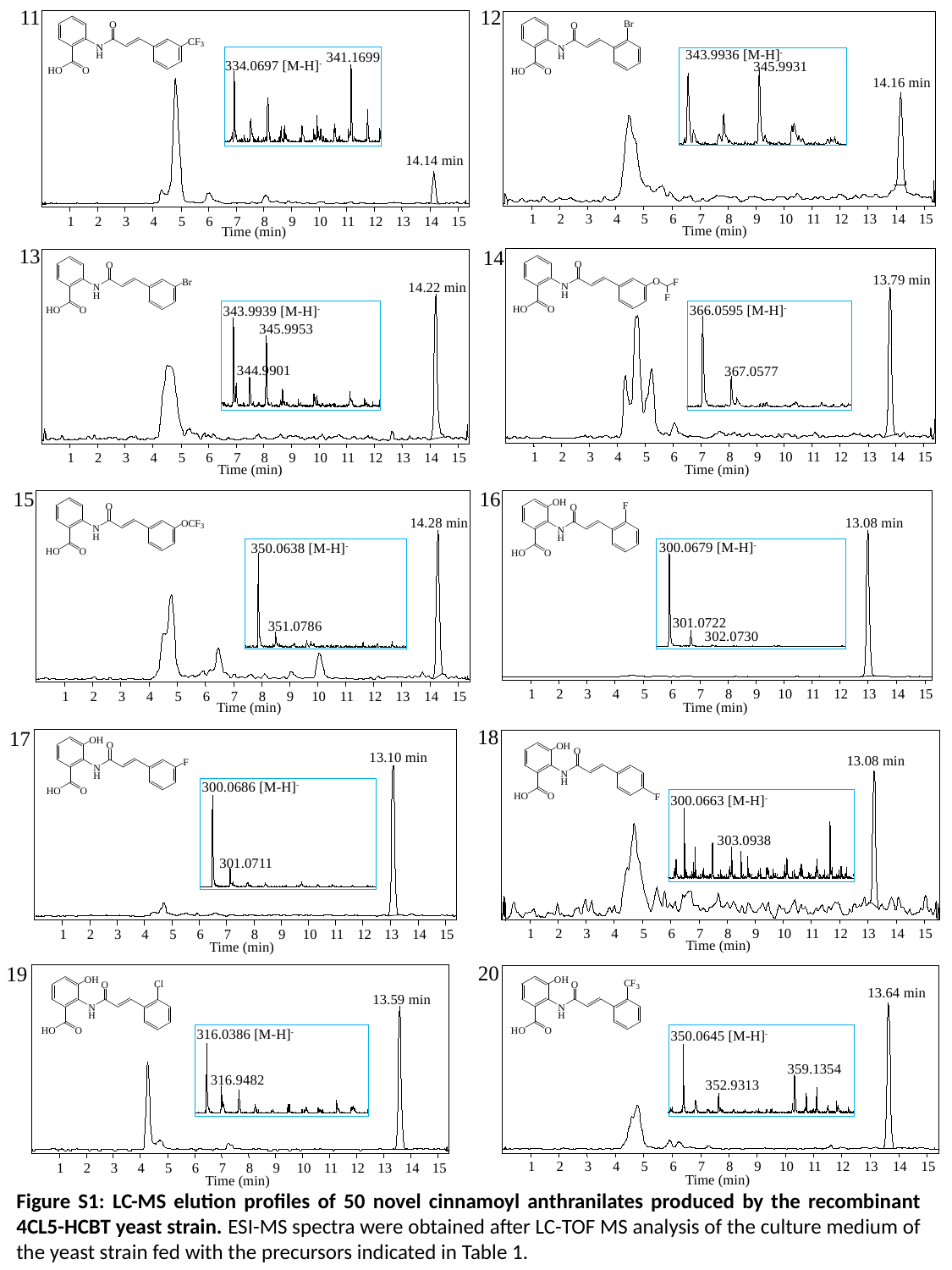

11
12
343.9936 [M-H]-
345.9931
14.16 min
1
2
3
4
5
6
7
8
9
10
11
12
13
14
15
Time (min)
341.1699
334.0697 [M-H]-
14.14 min
1
2
3
4
5
6
7
8
9
10
11
12
13
14
15
Time (min)
13
14
13.79 min
366.0595 [M-H]-
367.0577
1
2
3
4
5
6
7
8
9
10
11
12
13
14
15
Time (min)
14.22 min
343.9939 [M-H]-
345.9953
344.9901
1
2
3
4
5
6
7
8
9
10
11
12
13
14
15
Time (min)
15
16
 13.08 min
300.0679 [M-H]-
301.0722
302.0730
1
2
3
4
5
6
7
8
9
10
11
12
13
14
15
Time (min)
14.28 min
350.0638 [M-H]-
351.0786
1
2
3
4
5
6
7
8
9
10
11
12
13
14
15
Time (min)
18
17
13.10 min
300.0686 [M-H]-
301.0711
1
2
3
4
5
6
7
8
9
10
11
12
13
14
15
Time (min)
13.08 min
300.0663 [M-H]-
303.0938
1
2
3
4
5
6
7
8
9
10
11
12
13
14
15
Time (min)
20
19
13.59 min
316.0386 [M-H]-
316.9482
1
2
3
4
5
6
7
8
9
10
11
12
13
14
15
Time (min)
13.64 min
350.0645 [M-H]-
359.1354
352.9313
1
2
3
4
5
6
7
8
9
10
11
12
13
14
15
Time (min)
Figure S1: LC-MS elution profiles of 50 novel cinnamoyl anthranilates produced by the recombinant 4CL5-HCBT yeast strain. ESI-MS spectra were obtained after LC-TOF MS analysis of the culture medium of the yeast strain fed with the precursors indicated in Table 1.

## Slide 3
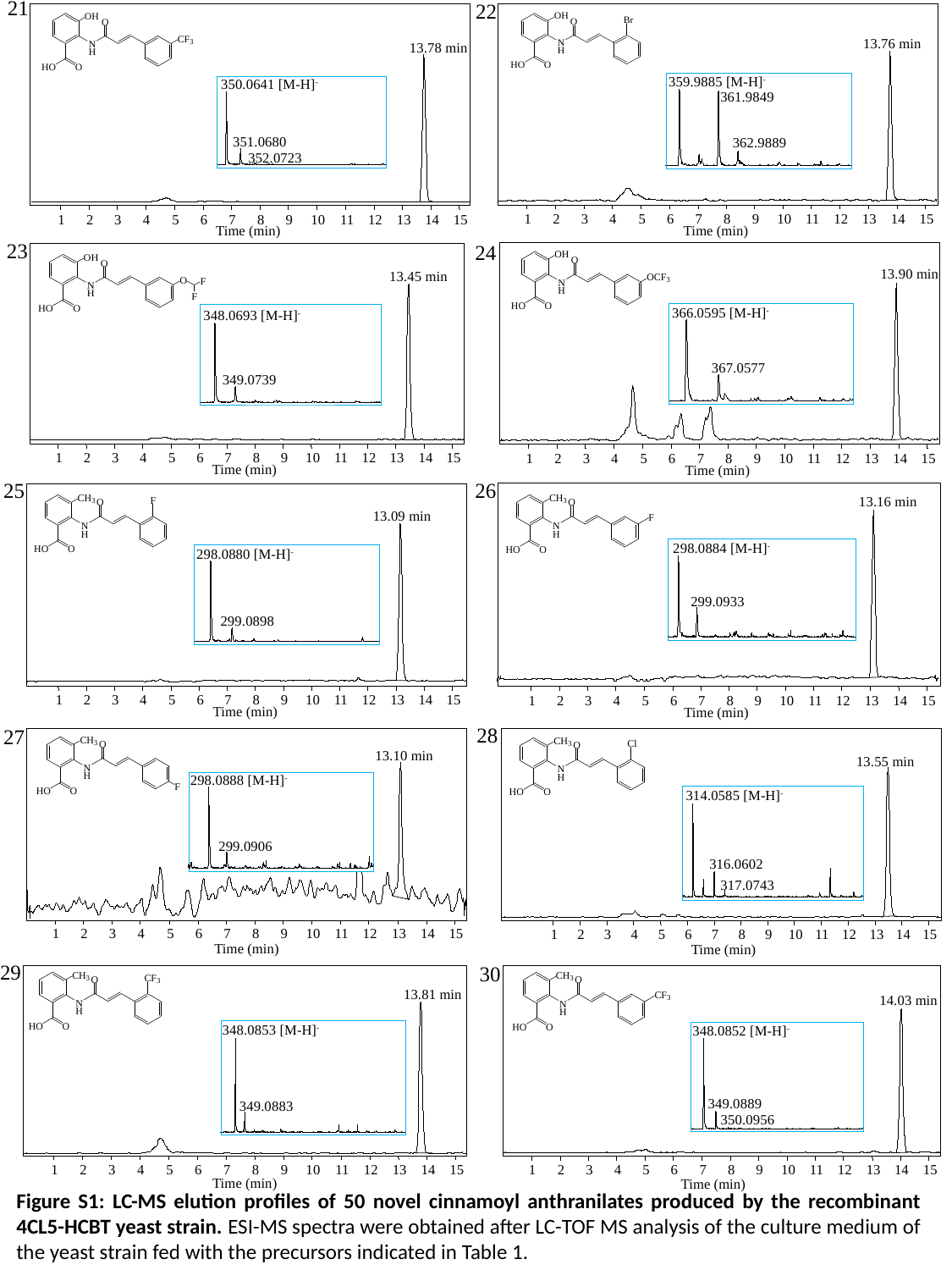

21
22
13.78 min
350.0641 [M-H]-
351.0680
352.0723
1
2
3
4
5
6
7
8
9
10
11
12
13
14
15
Time (min)
13.76 min
359.9885 [M-H]-
361.9849
362.9889
1
2
3
4
5
6
7
8
9
10
11
12
13
14
15
Time (min)
23
24
13.45 min
348.0693 [M-H]-
349.0739
1
2
3
4
5
6
7
8
9
10
11
12
13
14
15
Time (min)
13.90 min
366.0595 [M-H]-
367.0577
1
2
3
4
5
6
7
8
9
10
11
12
13
14
15
Time (min)
25
26
13.16 min
298.0884 [M-H]-
299.0933
1
2
3
4
5
6
7
8
9
10
11
12
13
14
15
Time (min)
13.09 min
298.0880 [M-H]-
299.0898
1
2
3
4
5
6
7
8
9
10
11
12
13
14
15
Time (min)
28
27
13.10 min
13.55 min
298.0888 [M-H]-
314.0585 [M-H]-
299.0906
316.0602
317.0743
1
2
3
4
5
6
7
8
9
10
11
12
13
14
15
1
2
3
4
5
6
7
8
9
10
11
12
13
14
15
Time (min)
Time (min)
29
30
13.81 min
14.03 min
348.0853 [M-H]-
348.0852 [M-H]-
349.0889
349.0883
350.0956
1
2
3
4
5
6
7
8
9
10
11
12
13
14
15
1
2
3
4
5
6
7
8
9
10
11
12
13
14
15
Time (min)
Time (min)
Figure S1: LC-MS elution profiles of 50 novel cinnamoyl anthranilates produced by the recombinant 4CL5-HCBT yeast strain. ESI-MS spectra were obtained after LC-TOF MS analysis of the culture medium of the yeast strain fed with the precursors indicated in Table 1.

## Slide 4
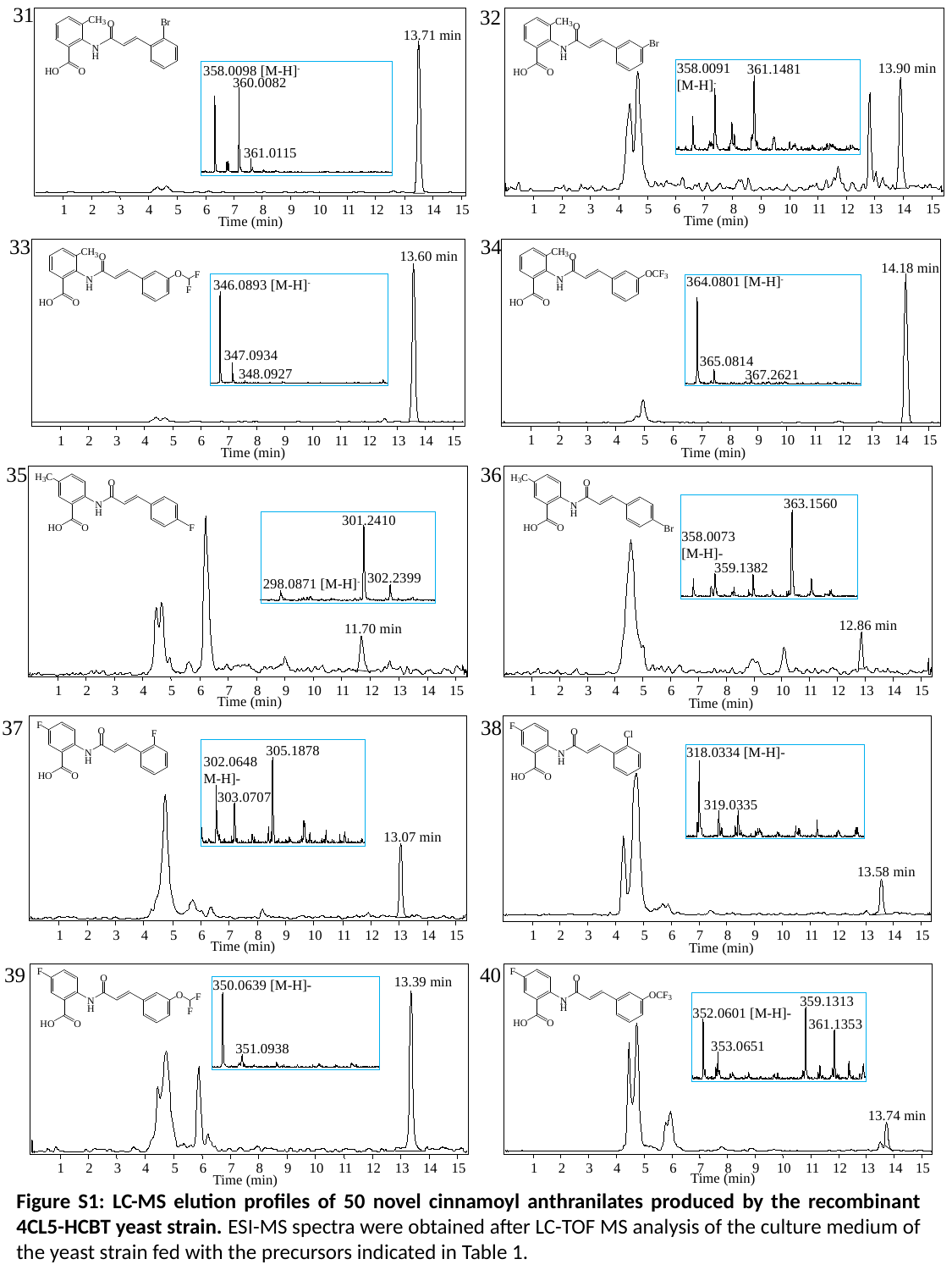

31
32
13.71 min
358.0098 [M-H]-
360.0082
361.0115
1
2
3
4
5
6
7
8
9
10
11
12
13
14
15
Time (min)
13.90 min
358.0091
[M-H]-
361.1481
1
2
3
4
5
6
7
8
9
10
11
12
13
14
15
Time (min)
34
33
 13.60 min
 14.18 min
364.0801 [M-H]-
346.0893 [M-H]-
347.0934
365.0814
348.0927
367.2621
1
2
3
4
5
6
7
8
9
10
11
12
13
14
15
1
2
3
4
5
6
7
8
9
10
11
12
13
14
15
Time (min)
Time (min)
36
35
363.1560
301.2410
358.0073
[M-H]-
359.1382
302.2399
298.0871 [M-H]-
 12.86 min
11.70 min
1
2
3
4
5
6
7
8
9
10
11
12
13
14
15
1
2
3
4
5
6
7
8
9
10
11
12
13
14
15
Time (min)
Time (min)
37
38
305.1878
318.0334 [M-H]-
302.0648
M-H]-
303.0707
319.0335
13.07 min
13.58 min
1
2
3
4
5
6
7
8
9
10
11
12
13
14
15
1
2
3
4
5
6
7
8
9
10
11
12
13
14
15
Time (min)
Time (min)
39
40
13.39 min
350.0639 [M-H]-
359.1313
352.0601 [M-H]-
361.1353
353.0651
351.0938
13.74 min
1
2
3
4
5
6
7
8
9
10
11
12
13
14
15
1
2
3
4
5
6
7
8
9
10
11
12
13
14
15
Time (min)
Time (min)
Figure S1: LC-MS elution profiles of 50 novel cinnamoyl anthranilates produced by the recombinant 4CL5-HCBT yeast strain. ESI-MS spectra were obtained after LC-TOF MS analysis of the culture medium of the yeast strain fed with the precursors indicated in Table 1.

## Slide 5
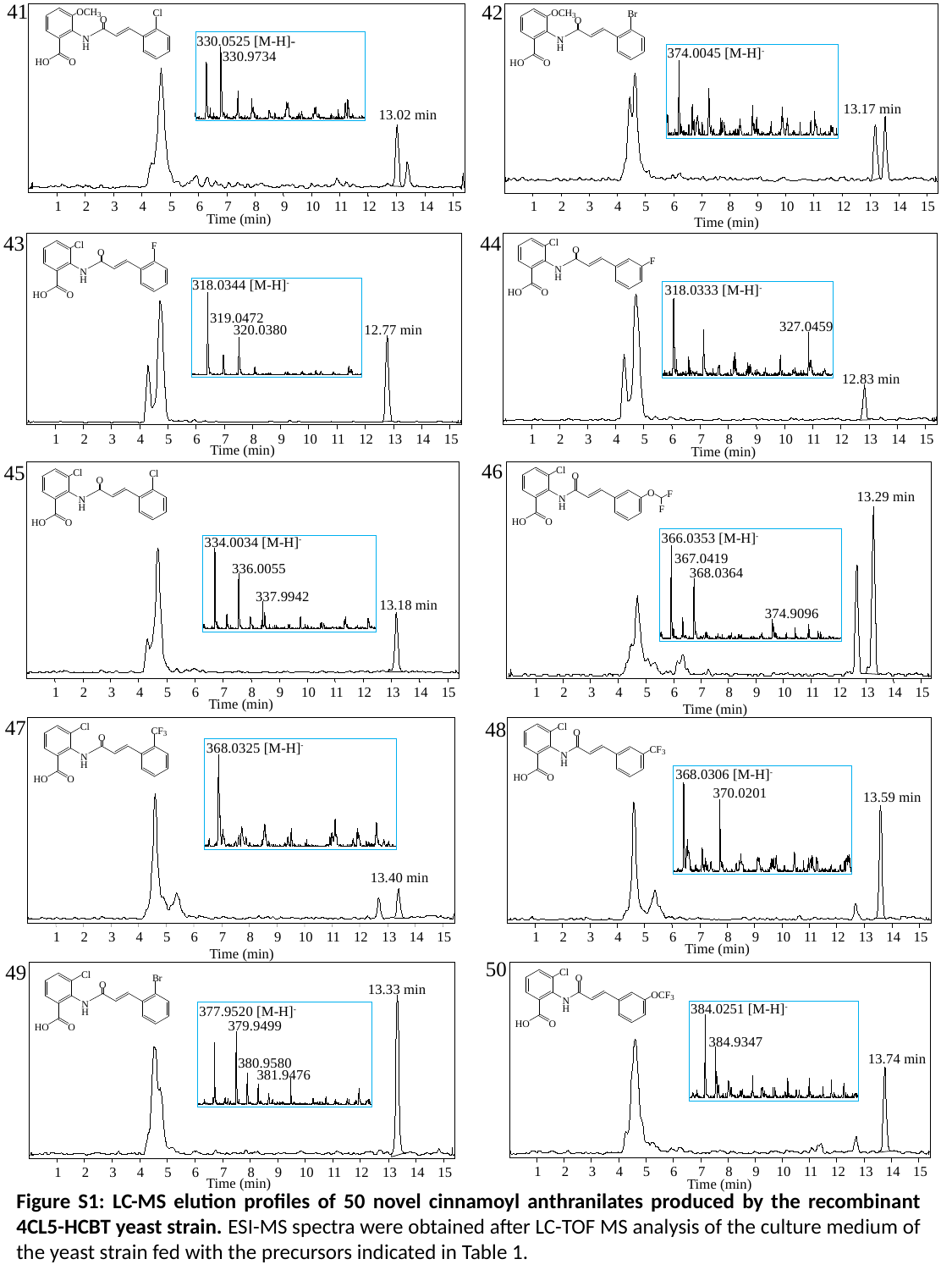

41
42
330.0525 [M-H]-
374.0045 [M-H]-
330.9734
13.17 min
13.02 min
1
2
3
4
5
6
7
8
9
10
11
12
13
14
15
1
2
3
4
5
6
7
8
9
10
11
12
13
14
15
Time (min)
Time (min)
43
44
318.0344 [M-H]-
318.0333 [M-H]-
319.0472
327.0459
12.77 min
320.0380
12.83 min
1
2
3
4
5
6
7
8
9
10
11
12
13
14
15
1
2
3
4
5
6
7
8
9
10
11
12
13
14
15
Time (min)
Time (min)
46
45
13.29 min
366.0353 [M-H]-
334.0034 [M-H]-
367.0419
336.0055
368.0364
337.9942
 13.18 min
374.9096
1
2
3
4
5
6
7
8
9
10
11
12
13
14
15
1
2
3
4
5
6
7
8
9
10
11
12
13
14
15
Time (min)
Time (min)
47
48
368.0325 [M-H]-
368.0306 [M-H]-
370.0201
13.59 min
13.40 min
1
2
3
4
5
6
7
8
9
10
11
12
13
14
15
1
2
3
4
5
6
7
8
9
10
11
12
13
14
15
Time (min)
Time (min)
50
49
13.33 min
384.0251 [M-H]-
377.9520 [M-H]-
379.9499
384.9347
13.74 min
380.9580
381.9476
1
2
3
4
5
6
7
8
9
10
11
12
13
14
15
1
2
3
4
5
6
7
8
9
10
11
12
13
14
15
Time (min)
Time (min)
Figure S1: LC-MS elution profiles of 50 novel cinnamoyl anthranilates produced by the recombinant 4CL5-HCBT yeast strain. ESI-MS spectra were obtained after LC-TOF MS analysis of the culture medium of the yeast strain fed with the precursors indicated in Table 1.
