# Supplementary material for: Precursor-Directed Combinatorial Biosynthesis of Cinnamoyl, Dihydrocinnamoyl, and Benzoyl Anthranilates in Saccharomyces cerevisiae
Source: PLoS One. 2015 Oct 2;10(10):e0138972. doi: 10.1371/journal.pone.0138972 (PMC4591981; doi:10.1371/journal.pone.0138972)
Supplement: S2 Fig — ESI-MS spectra were obtained after LC-TOF MS analysis of the culture medium of the yeast strain fed with the precursors indicated in Table 2. (PPTX) [file pone.0138972.s002.pptx]

## Slide 1
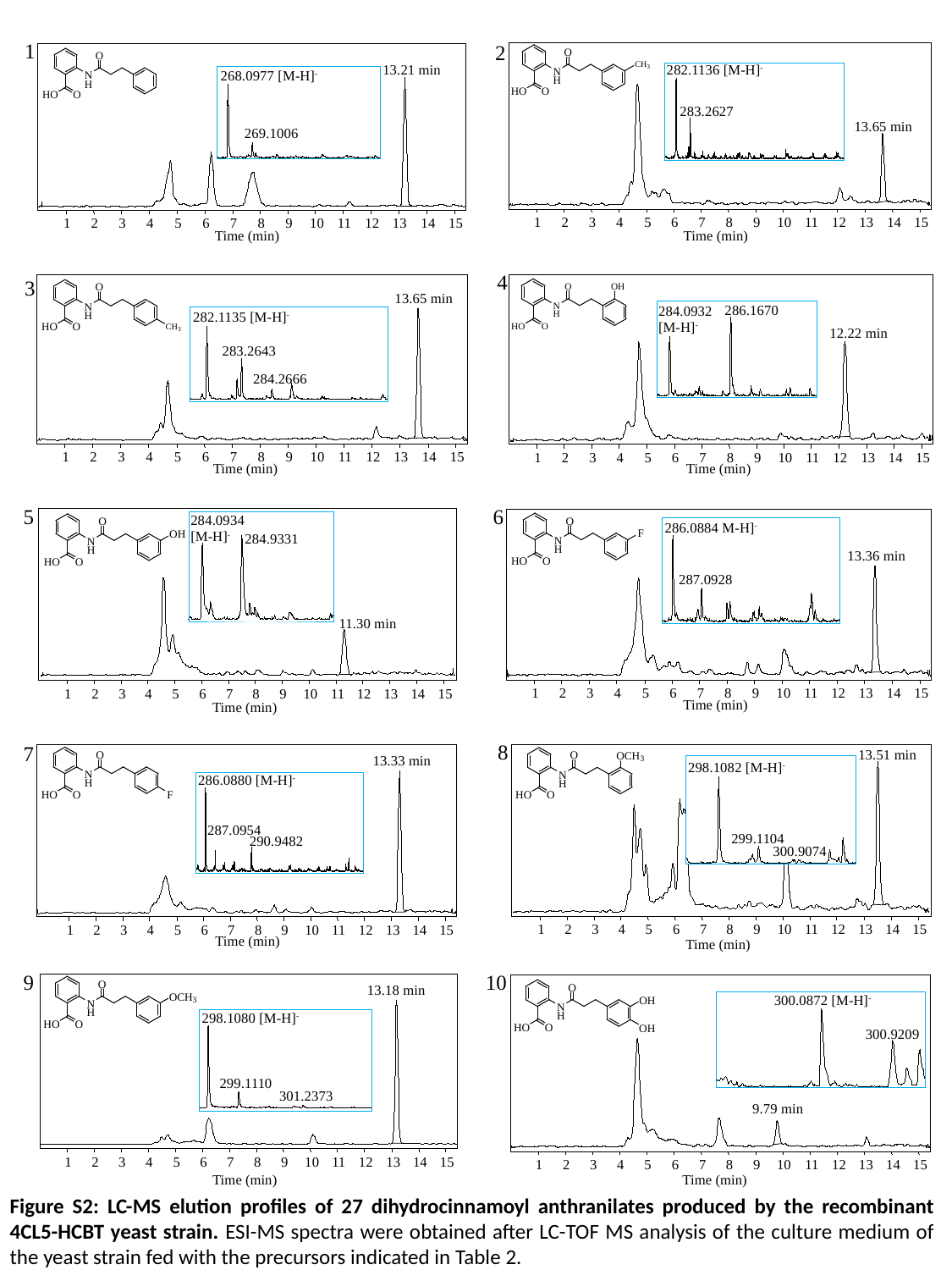

1
2
282.1136 [M-H]-
13.21 min
268.0977 [M-H]-
283.2627
13.65 min
269.1006
1
2
3
4
5
6
7
8
9
10
11
12
13
14
15
1
2
3
4
5
6
7
8
9
10
11
12
13
14
15
Time (min)
Time (min)
4
3
13.65 min
286.1670
284.0932
[M-H]-
282.1135 [M-H]-
12.22 min
283.2643
284.2666
1
2
3
4
5
6
7
8
9
10
11
12
13
14
15
1
2
3
4
5
6
7
8
9
10
11
12
13
14
15
Time (min)
Time (min)
5
6
284.0934
[M-H]-
286.0884 M-H]-
284.9331
13.36 min
287.0928
11.30 min
1
2
3
4
5
6
7
8
9
10
11
12
13
14
15
1
2
3
4
5
6
7
8
9
10
11
12
13
14
15
Time (min)
Time (min)
8
7
13.51 min
13.33 min
298.1082 [M-H]-
286.0880 [M-H]-
287.0954
299.1104
290.9482
300.9074
1
2
3
4
5
6
7
8
9
10
11
12
13
14
15
1
2
3
4
5
6
7
8
9
10
11
12
13
14
15
Time (min)
Time (min)
9
10
13.18 min
300.0872 [M-H]-
298.1080 [M-H]-
300.9209
299.1110
301.2373
9.79 min
1
2
3
4
5
6
7
8
9
10
11
12
13
14
15
1
2
3
4
5
6
7
8
9
10
11
12
13
14
15
Time (min)
Time (min)
Figure S2: LC-MS elution profiles of 27 dihydrocinnamoyl anthranilates produced by the recombinant 4CL5-HCBT yeast strain. ESI-MS spectra were obtained after LC-TOF MS analysis of the culture medium of the yeast strain fed with the precursors indicated in Table 2.

## Slide 2
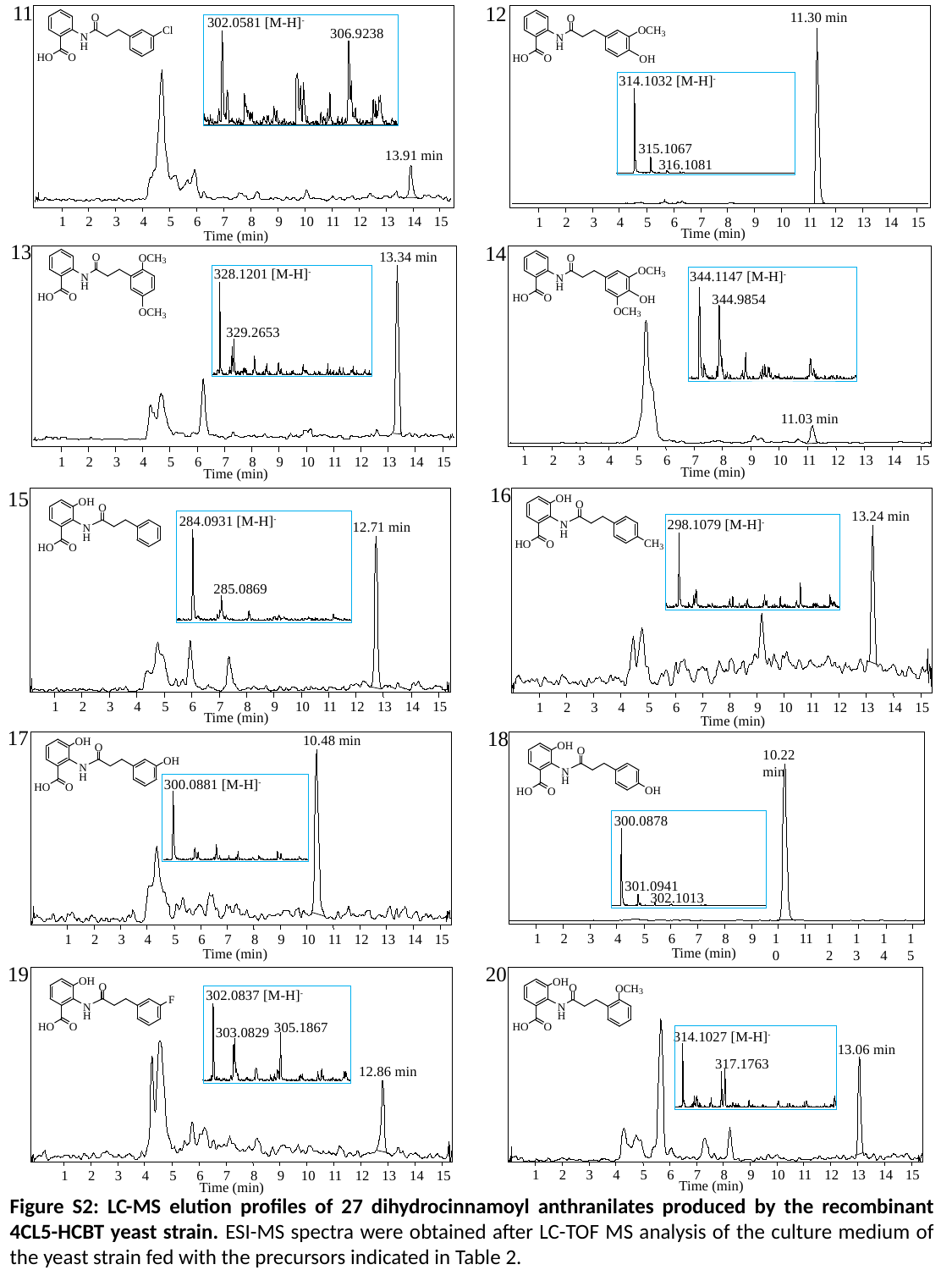

11
12
11.30 min
302.0581 [M-H]-
306.9238
314.1032 [M-H]-
315.1067
13.91 min
316.1081
1
2
3
4
5
6
7
8
9
10
11
12
13
14
15
1
2
3
4
5
6
7
8
9
10
11
12
13
14
15
Time (min)
Time (min)
13
14
13.34 min
328.1201 [M-H]-
344.1147 [M-H]-
344.9854
329.2653
11.03 min
1
2
3
4
5
6
7
8
9
10
11
12
13
14
15
1
2
3
4
5
6
7
8
9
10
11
12
13
14
15
Time (min)
Time (min)
16
15
13.24 min
284.0931 [M-H]-
298.1079 [M-H]-
12.71 min
285.0869
1
2
3
4
5
6
7
8
9
10
11
12
13
14
15
1
2
3
4
5
6
7
8
9
10
11
12
13
14
15
Time (min)
Time (min)
17
18
10.48 min
10.22 min
300.0881 [M-H]-
300.0878
301.0941
302.1013
1
2
3
4
5
6
7
8
9
10
11
12
13
14
15
1
2
3
4
5
6
7
8
9
10
11
12
13
14
15
Time (min)
Time (min)
19
20
314.1027 [M-H]-
 13.06 min
317.1763
1
2
3
4
5
6
7
8
9
10
11
12
13
14
15
Time (min)
302.0837 [M-H]-
305.1867
303.0829
12.86 min
1
2
3
4
5
6
7
8
9
10
11
12
13
14
15
Time (min)
Figure S2: LC-MS elution profiles of 27 dihydrocinnamoyl anthranilates produced by the recombinant 4CL5-HCBT yeast strain. ESI-MS spectra were obtained after LC-TOF MS analysis of the culture medium of the yeast strain fed with the precursors indicated in Table 2.

## Slide 3
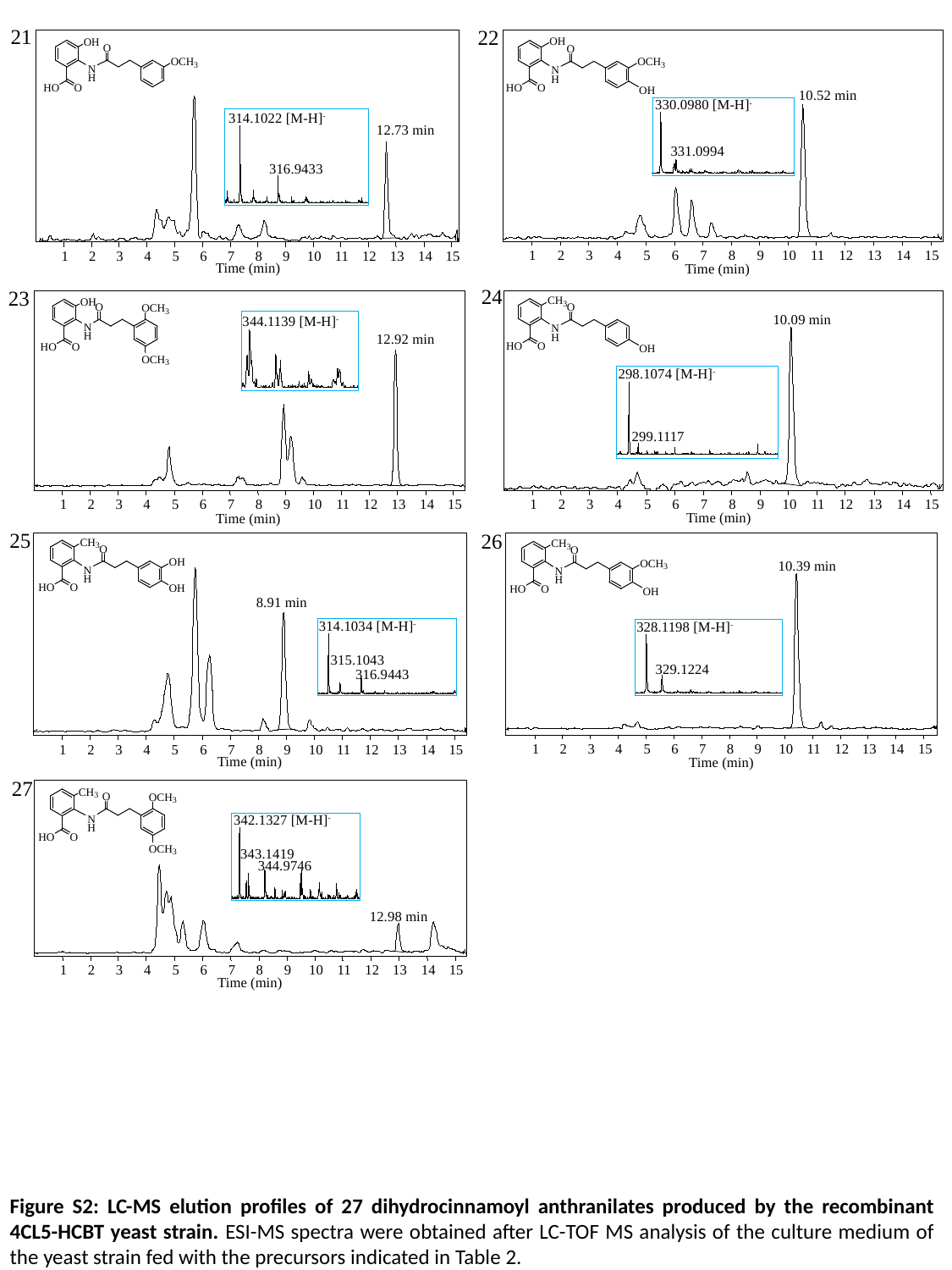

21
22
314.1022 [M-H]-
12.73 min
316.9433
1
2
3
4
5
6
7
8
9
10
11
12
13
14
15
Time (min)
10.52 min
330.0980 [M-H]-
331.0994
1
2
3
4
5
6
7
8
9
10
11
12
13
14
15
Time (min)
24
23
10.09 min
344.1139 [M-H]-
12.92 min
298.1074 [M-H]-
299.1117
1
2
3
4
5
6
7
8
9
10
11
12
13
14
15
1
2
3
4
5
6
7
8
9
10
11
12
13
14
15
Time (min)
Time (min)
25
26
10.39 min
8.91 min
314.1034 [M-H]-
328.1198 [M-H]-
315.1043
329.1224
316.9443
1
2
3
4
5
6
7
8
9
10
11
12
13
14
15
1
2
3
4
5
6
7
8
9
10
11
12
13
14
15
Time (min)
Time (min)
27
342.1327 [M-H]-
343.1419
344.9746
12.98 min
1
2
3
4
5
6
7
8
9
10
11
12
13
14
15
Time (min)
Figure S2: LC-MS elution profiles of 27 dihydrocinnamoyl anthranilates produced by the recombinant 4CL5-HCBT yeast strain. ESI-MS spectra were obtained after LC-TOF MS analysis of the culture medium of the yeast strain fed with the precursors indicated in Table 2.
