# Supplementary material for: Precursor-Directed Combinatorial Biosynthesis of Cinnamoyl, Dihydrocinnamoyl, and Benzoyl Anthranilates in Saccharomyces cerevisiae
Source: PLoS One. 2015 Oct 2;10(10):e0138972. doi: 10.1371/journal.pone.0138972 (PMC4591981; doi:10.1371/journal.pone.0138972)
Supplement: S3 Fig — ESI-MS spectra were obtained after LC-TOF MS analysis of the culture medium of the yeast strain fed with the precursors indicated in Table 3. (PPTX) [file pone.0138972.s003.pptx]

## Slide 1
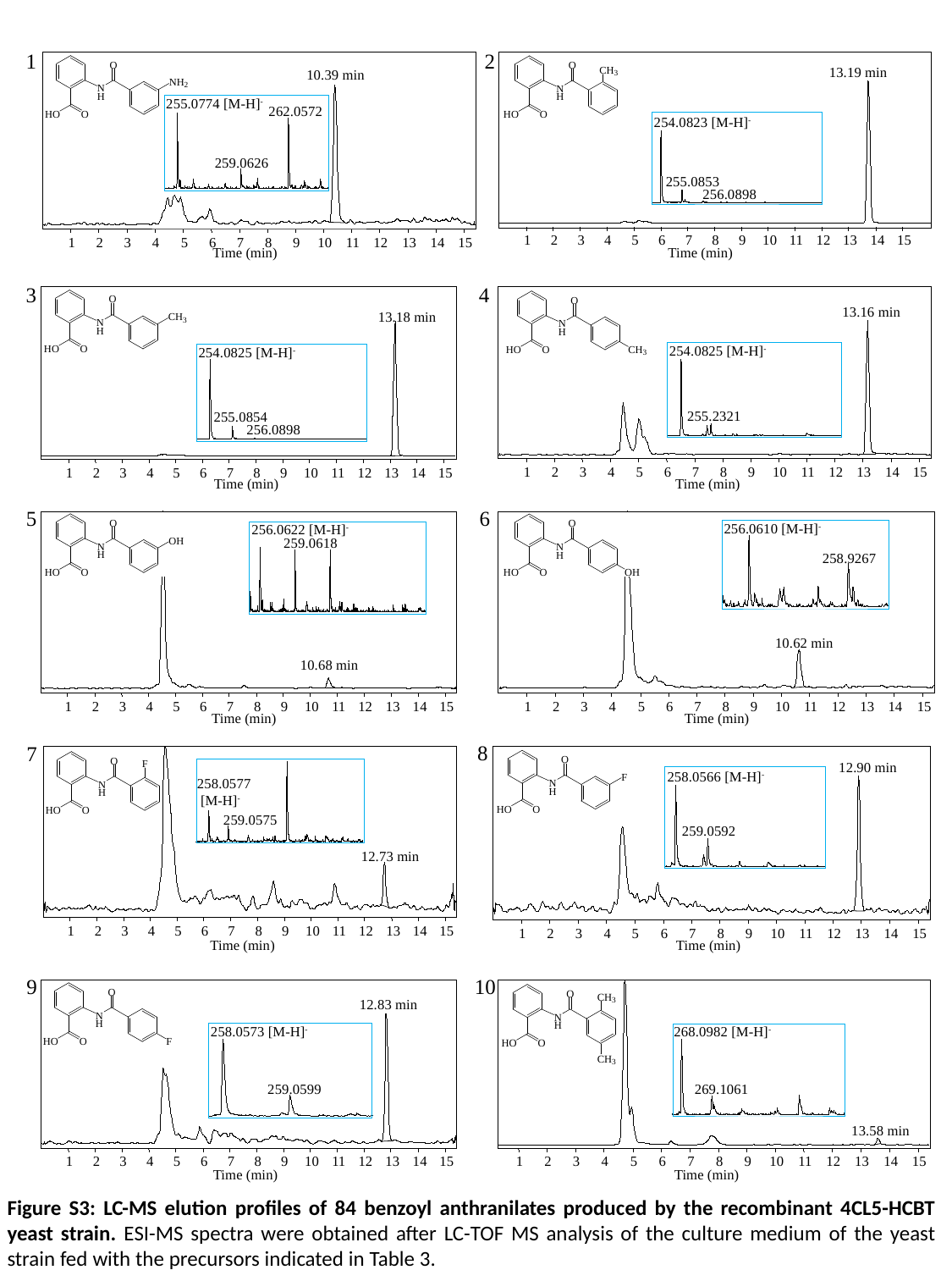

2
1
13.19 min
10.39 min
255.0774 [M-H]-
262.0572
254.0823 [M-H]-
259.0626
255.0853
256.0898
1
2
3
4
5
6
7
8
9
10
11
12
13
14
15
1
2
3
4
5
6
7
8
9
10
11
12
13
14
15
Time (min)
Time (min)
3
4
13.16 min
13.18 min
254.0825 [M-H]-
254.0825 [M-H]-
255.2321
255.0854
256.0898
1
2
3
4
5
6
7
8
9
10
11
12
13
14
15
1
2
3
4
5
6
7
8
9
10
11
12
13
14
15
Time (min)
Time (min)
5
6
256.0610 [M-H]-
256.0622 [M-H]-
259.0618
258.9267
10.62 min
10.68 min
1
2
3
4
5
6
7
8
9
10
11
12
13
14
15
1
2
3
4
5
6
7
8
9
10
11
12
13
14
15
Time (min)
Time (min)
8
7
12.90 min
258.0566 [M-H]-
258.0577
 [M-H]-
259.0575
259.0592
12.73 min
1
2
3
4
5
6
7
8
9
10
11
12
13
14
15
1
2
3
4
5
6
7
8
9
10
11
12
13
14
15
Time (min)
Time (min)
9
10
12.83 min
258.0573 [M-H]-
268.0982 [M-H]-
259.0599
269.1061
13.58 min
1
2
3
4
5
6
7
8
9
10
11
12
13
14
15
1
2
3
4
5
6
7
8
9
10
11
12
13
14
15
Time (min)
Time (min)
Figure S3: LC-MS elution profiles of 84 benzoyl anthranilates produced by the recombinant 4CL5-HCBT yeast strain. ESI-MS spectra were obtained after LC-TOF MS analysis of the culture medium of the yeast strain fed with the precursors indicated in Table 3.

## Slide 2
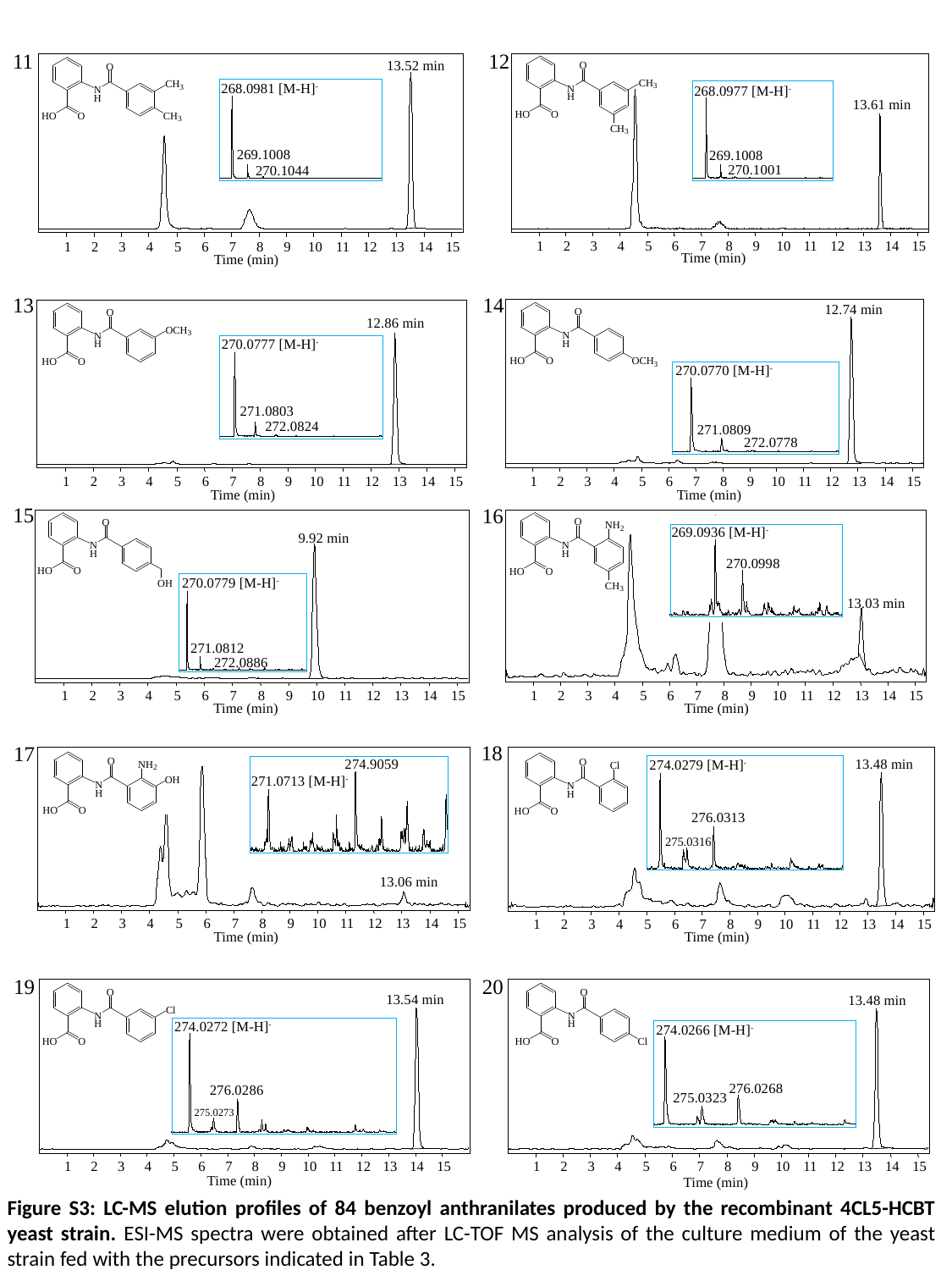

12
11
13.52 min
268.0981 [M-H]-
268.0977 [M-H]-
13.61 min
269.1008
269.1008
270.1001
270.1044
1
2
3
4
5
6
7
8
9
10
11
12
13
14
15
1
2
3
4
5
6
7
8
9
10
11
12
13
14
15
Time (min)
Time (min)
13
14
12.74 min
12.86 min
270.0777 [M-H]-
270.0770 [M-H]-
271.0803
272.0824
271.0809
272.0778
1
2
3
4
5
6
7
8
9
10
11
12
13
14
15
1
2
3
4
5
6
7
8
9
10
11
12
13
14
15
Time (min)
Time (min)
15
16
269.0936 [M-H]-
9.92 min
270.0998
270.0779 [M-H]-
13.03 min
271.0812
272.0886
1
2
3
4
5
6
7
8
9
10
11
12
13
14
15
1
2
3
4
5
6
7
8
9
10
11
12
13
14
15
Time (min)
Time (min)
18
17
13.48 min
274.9059
274.0279 [M-H]-
271.0713 [M-H]-
276.0313
275.0316
13.06 min
1
2
3
4
5
6
7
8
9
10
11
12
13
14
15
1
2
3
4
5
6
7
8
9
10
11
12
13
14
15
Time (min)
Time (min)
19
20
13.54 min
13.48 min
274.0272 [M-H]-
274.0266 [M-H]-
276.0268
276.0286
275.0323
275.0273
1
2
3
4
5
6
7
8
9
10
11
12
13
14
15
1
2
3
4
5
6
7
8
9
10
11
12
13
14
15
Time (min)
Time (min)
Figure S3: LC-MS elution profiles of 84 benzoyl anthranilates produced by the recombinant 4CL5-HCBT yeast strain. ESI-MS spectra were obtained after LC-TOF MS analysis of the culture medium of the yeast strain fed with the precursors indicated in Table 3.

## Slide 3
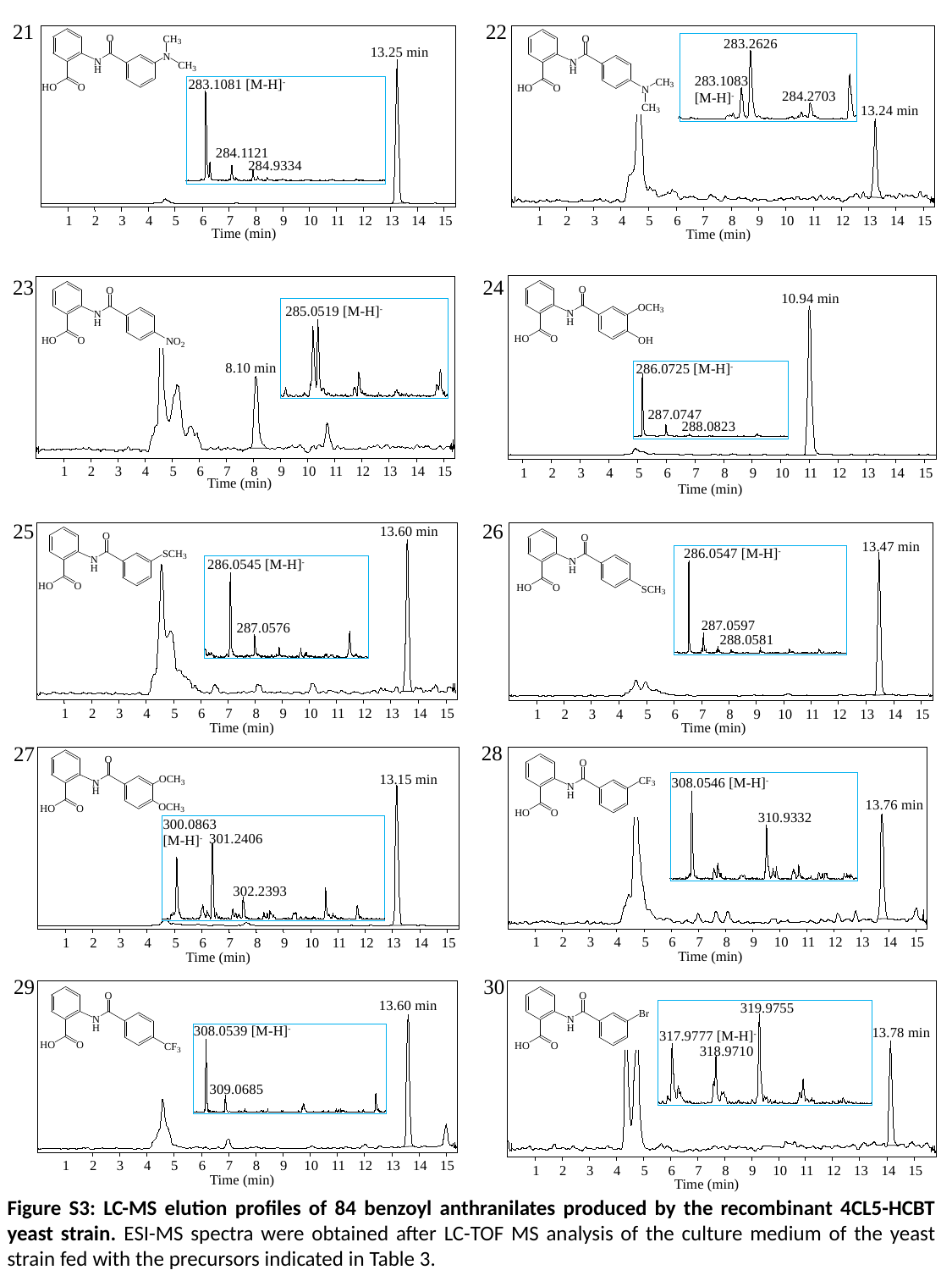

22
21
283.2626
13.25 min
283.1083
[M-H]-
283.1081 [M-H]-
284.2703
13.24 min
284.1121
284.9334
1
2
3
4
5
6
7
8
9
10
11
12
13
14
15
1
2
3
4
5
6
7
8
9
10
11
12
13
14
15
Time (min)
Time (min)
23
24
10.94 min
285.0519 [M-H]-
8.10 min
286.0725 [M-H]-
287.0747
288.0823
1
2
3
4
5
6
7
8
9
10
11
12
13
14
15
1
2
3
4
5
6
7
8
9
10
11
12
13
14
15
Time (min)
Time (min)
25
26
13.60 min
13.47 min
286.0547 [M-H]-
286.0545 [M-H]-
287.0597
287.0576
288.0581
1
2
3
4
5
6
7
8
9
10
11
12
13
14
15
1
2
3
4
5
6
7
8
9
10
11
12
13
14
15
Time (min)
Time (min)
28
27
13.15 min
308.0546 [M-H]-
13.76 min
310.9332
300.0863
[M-H]-
301.2406
302.2393
1
2
3
4
5
6
7
8
9
10
11
12
13
14
15
1
2
3
4
5
6
7
8
9
10
11
12
13
14
15
Time (min)
Time (min)
29
30
13.60 min
319.9755
308.0539 [M-H]-
13.78 min
317.9777 [M-H]-
318.9710
309.0685
1
2
3
4
5
6
7
8
9
10
11
12
13
14
15
1
2
3
4
5
6
7
8
9
10
11
12
13
14
15
Time (min)
Time (min)
Figure S3: LC-MS elution profiles of 84 benzoyl anthranilates produced by the recombinant 4CL5-HCBT yeast strain. ESI-MS spectra were obtained after LC-TOF MS analysis of the culture medium of the yeast strain fed with the precursors indicated in Table 3.

## Slide 4
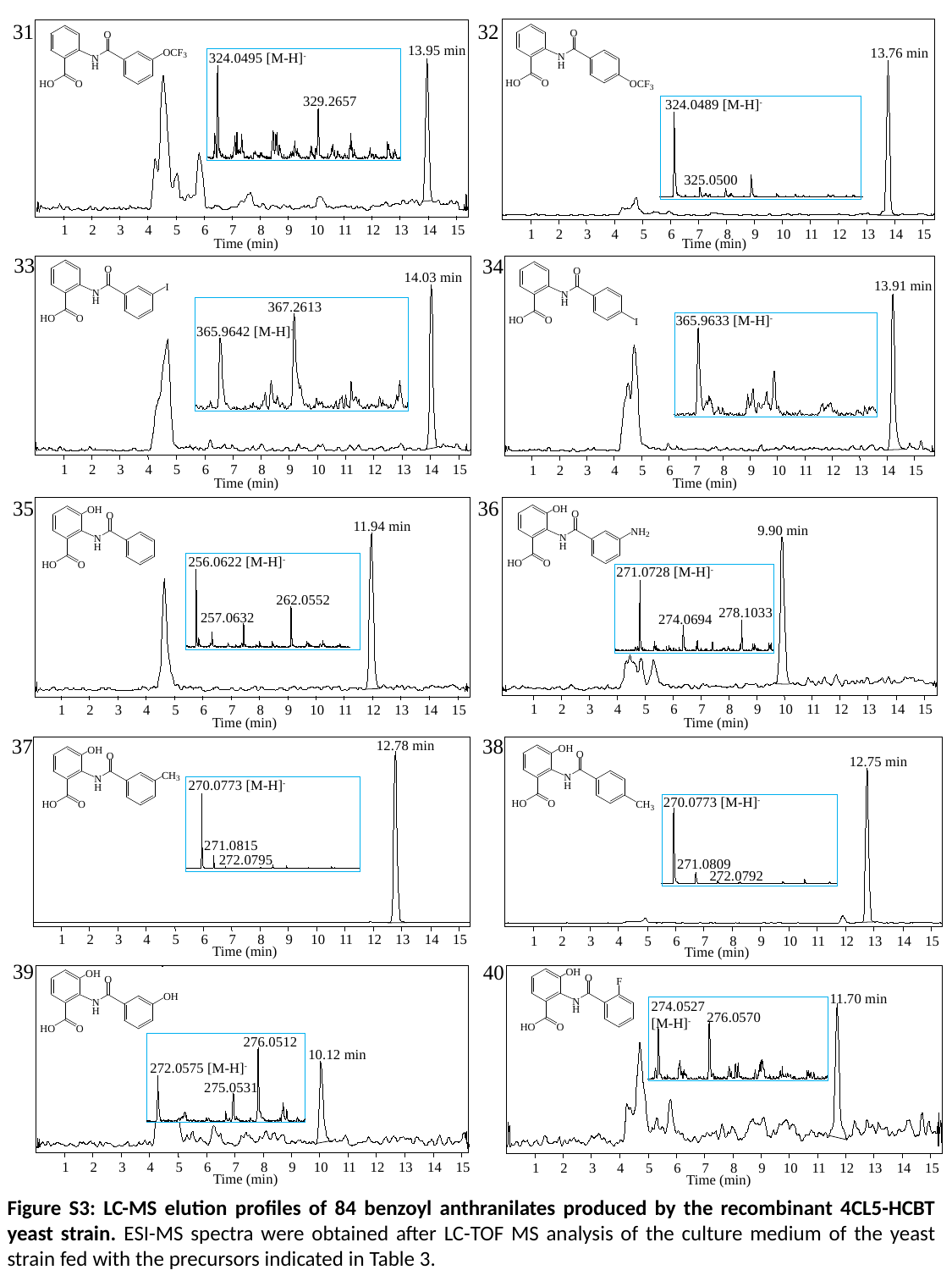

32
31
13.95 min
13.76 min
324.0495 [M-H]-
329.2657
324.0489 [M-H]-
325.0500
1
2
3
4
5
6
7
8
9
10
11
12
13
14
15
1
2
3
4
5
6
7
8
9
10
11
12
13
14
15
Time (min)
Time (min)
33
34
14.03 min
13.91 min
367.2613
365.9633 [M-H]-
365.9642 [M-H]-
1
2
3
4
5
6
7
8
9
10
11
12
13
14
15
1
2
3
4
5
6
7
8
9
10
11
12
13
14
15
Time (min)
Time (min)
36
35
11.94 min
9.90 min
256.0622 [M-H]-
271.0728 [M-H]-
262.0552
278.1033
257.0632
274.0694
1
2
3
4
5
6
7
8
9
10
11
12
13
14
15
1
2
3
4
5
6
7
8
9
10
11
12
13
14
15
Time (min)
Time (min)
37
38
12.75 min
270.0773 [M-H]-
271.0809
272.0792
1
2
3
4
5
6
7
8
9
10
11
12
13
14
15
Time (min)
12.78 min
270.0773 [M-H]-
271.0815
272.0795
1
2
3
4
5
6
7
8
9
10
11
12
13
14
15
Time (min)
39
40
11.70 min
274.0527
[M-H]-
276.0570
276.0512
10.12 min
272.0575 [M-H]-
275.0531
1
2
3
4
5
6
7
8
9
10
11
12
13
14
15
1
2
3
4
5
6
7
8
9
10
11
12
13
14
15
Time (min)
Time (min)
Figure S3: LC-MS elution profiles of 84 benzoyl anthranilates produced by the recombinant 4CL5-HCBT yeast strain. ESI-MS spectra were obtained after LC-TOF MS analysis of the culture medium of the yeast strain fed with the precursors indicated in Table 3.

## Slide 5
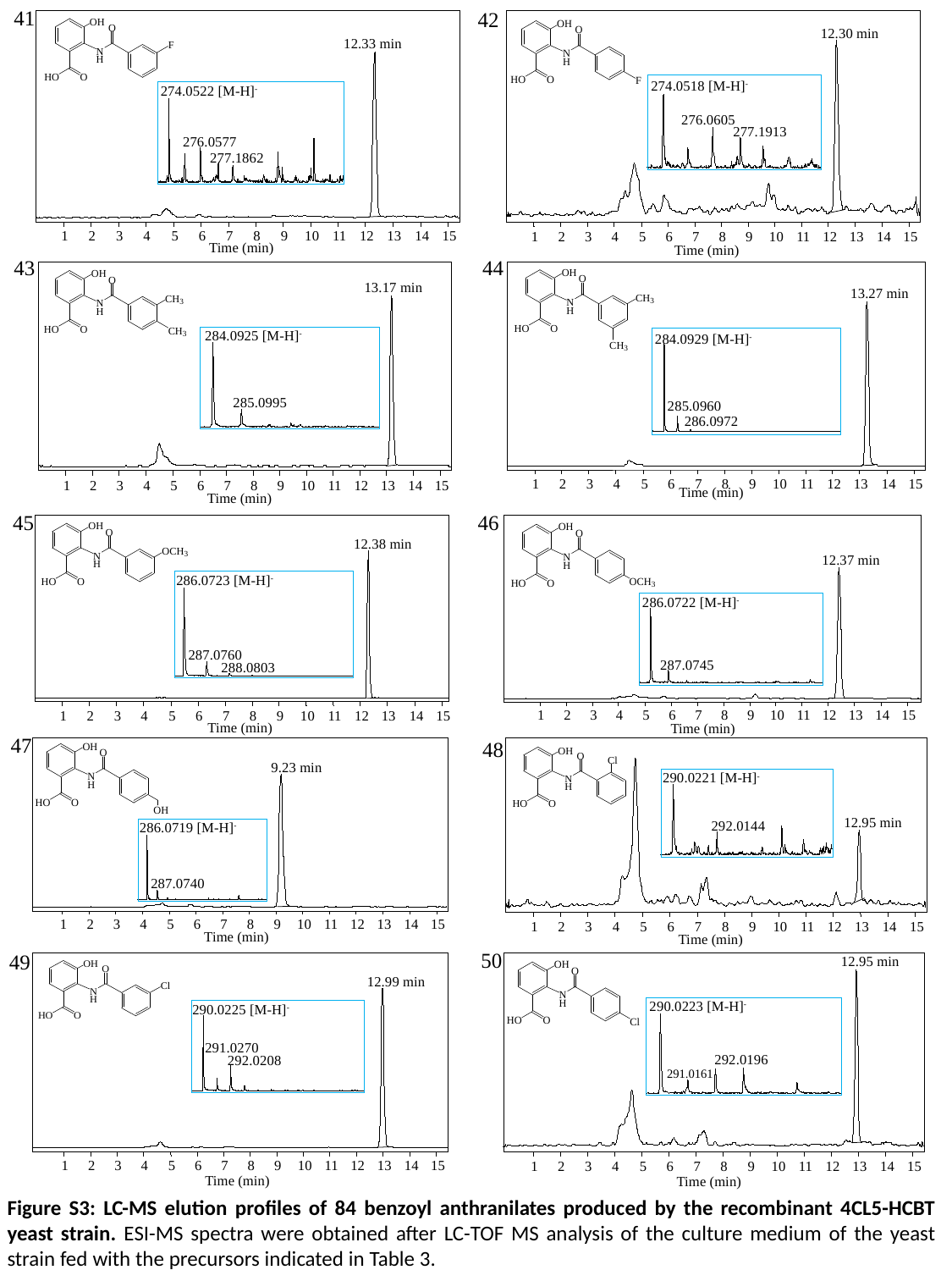

41
42
12.33 min
274.0522 [M-H]-
276.0577
277.1862
1
2
3
4
5
6
7
8
9
10
11
12
13
14
15
Time (min)
12.30 min
274.0518 [M-H]-
276.0605
277.1913
1
2
3
4
5
6
7
8
9
10
11
12
13
14
15
Time (min)
43
44
13.17 min
284.0925 [M-H]-
285.0995
1
2
3
4
5
6
7
8
9
10
11
12
13
14
15
Time (min)
13.27 min
284.0929 [M-H]-
285.0960
286.0972
1
2
3
4
5
6
7
8
9
10
11
12
13
14
15
Time (min)
46
45
12.38 min
12.37 min
286.0723 [M-H]-
286.0722 [M-H]-
287.0760
287.0745
288.0803
1
2
3
4
5
6
7
8
9
10
11
12
13
14
15
1
2
3
4
5
6
7
8
9
10
11
12
13
14
15
Time (min)
Time (min)
47
48
290.0221 [M-H]-
12.95 min
292.0144
1
2
3
4
5
6
7
8
9
10
11
12
13
14
15
Time (min)
9.23 min
286.0719 [M-H]-
287.0740
1
2
3
4
5
6
7
8
9
10
11
12
13
14
15
Time (min)
50
49
12.95 min
290.0223 [M-H]-
292.0196
291.0161
1
2
3
4
5
6
7
8
9
10
11
12
13
14
15
Time (min)
12.99 min
290.0225 [M-H]-
291.0270
292.0208
1
2
3
4
5
6
7
8
9
10
11
12
13
14
15
Time (min)
Figure S3: LC-MS elution profiles of 84 benzoyl anthranilates produced by the recombinant 4CL5-HCBT yeast strain. ESI-MS spectra were obtained after LC-TOF MS analysis of the culture medium of the yeast strain fed with the precursors indicated in Table 3.

## Slide 6
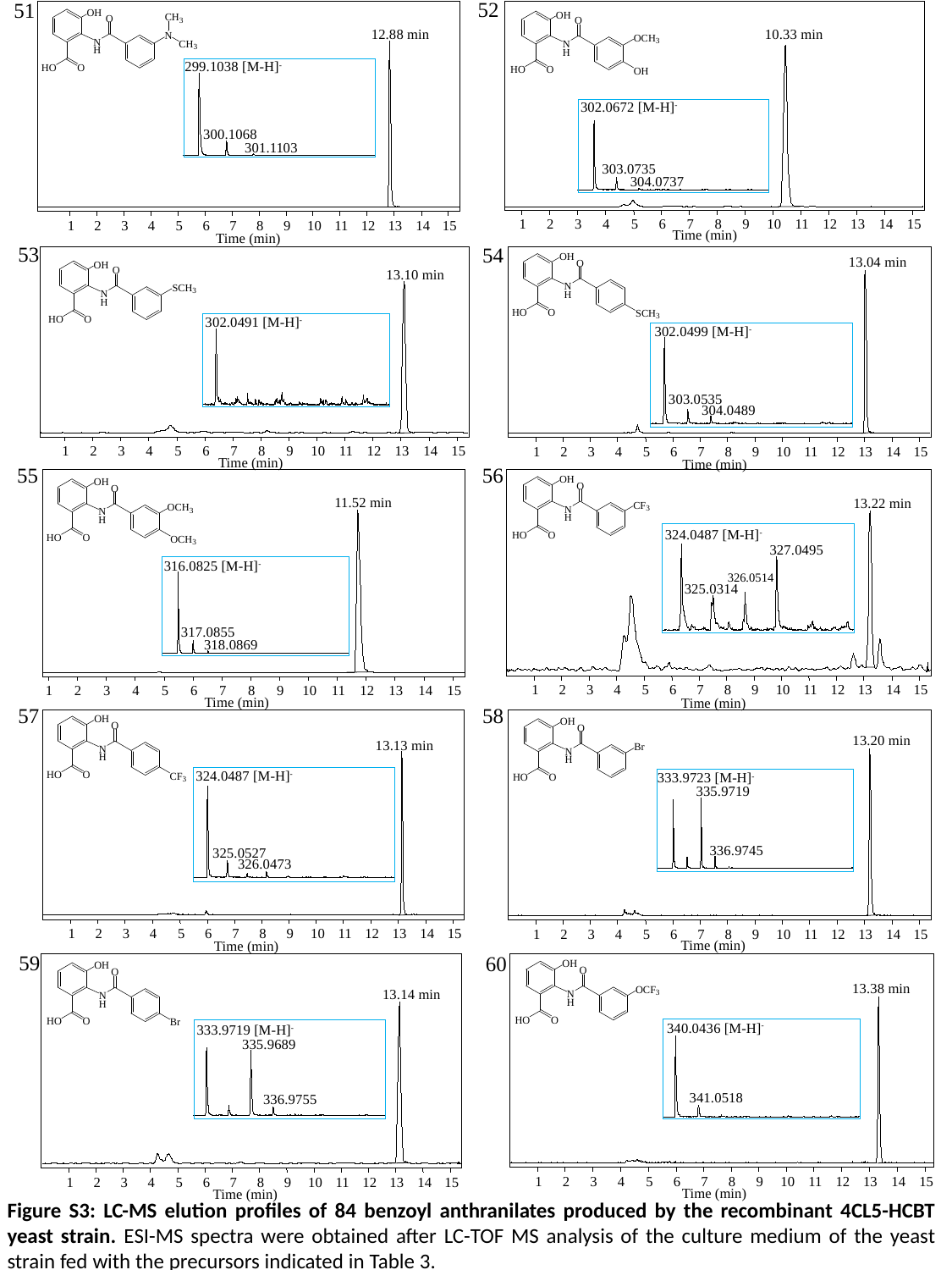

52
51
12.88 min
299.1038 [M-H]-
300.1068
301.1103
1
2
3
4
5
6
7
8
9
10
11
12
13
14
15
Time (min)
10.33 min
302.0672 [M-H]-
303.0735
304.0737
1
2
3
4
5
6
7
8
9
10
11
12
13
14
15
Time (min)
53
54
13.10 min
302.0491 [M-H]-
1
2
3
4
5
6
7
8
9
10
11
12
13
14
15
Time (min)
13.04 min
302.0499 [M-H]-
303.0535
304.0489
1
2
3
4
5
6
7
8
9
10
11
12
13
14
15
56
55
Time (min)
13.22 min
324.0487 [M-H]-
327.0495
326.0514
325.0314
1
2
3
4
5
6
7
8
9
10
11
12
13
14
15
Time (min)
11.52 min
316.0825 [M-H]-
317.0855
318.0869
1
2
3
4
5
6
7
8
9
10
11
12
13
14
15
Time (min)
57
58
13.13 min
324.0487 [M-H]-
325.0527
326.0473
1
2
3
4
5
6
7
8
9
10
11
12
13
14
15
Time (min)
13.20 min
333.9723 [M-H]-
335.9719
336.9745
1
2
3
4
5
6
7
8
9
10
11
12
13
14
15
Time (min)
59
60
13.14 min
333.9719 [M-H]-
335.9689
336.9755
1
2
3
4
5
6
7
8
9
10
11
12
13
14
15
Time (min)
13.38 min
340.0436 [M-H]-
341.0518
1
2
3
4
5
6
7
8
9
10
11
12
13
14
15
Time (min)
Figure S3: LC-MS elution profiles of 84 benzoyl anthranilates produced by the recombinant 4CL5-HCBT yeast strain. ESI-MS spectra were obtained after LC-TOF MS analysis of the culture medium of the yeast strain fed with the precursors indicated in Table 3.

## Slide 7
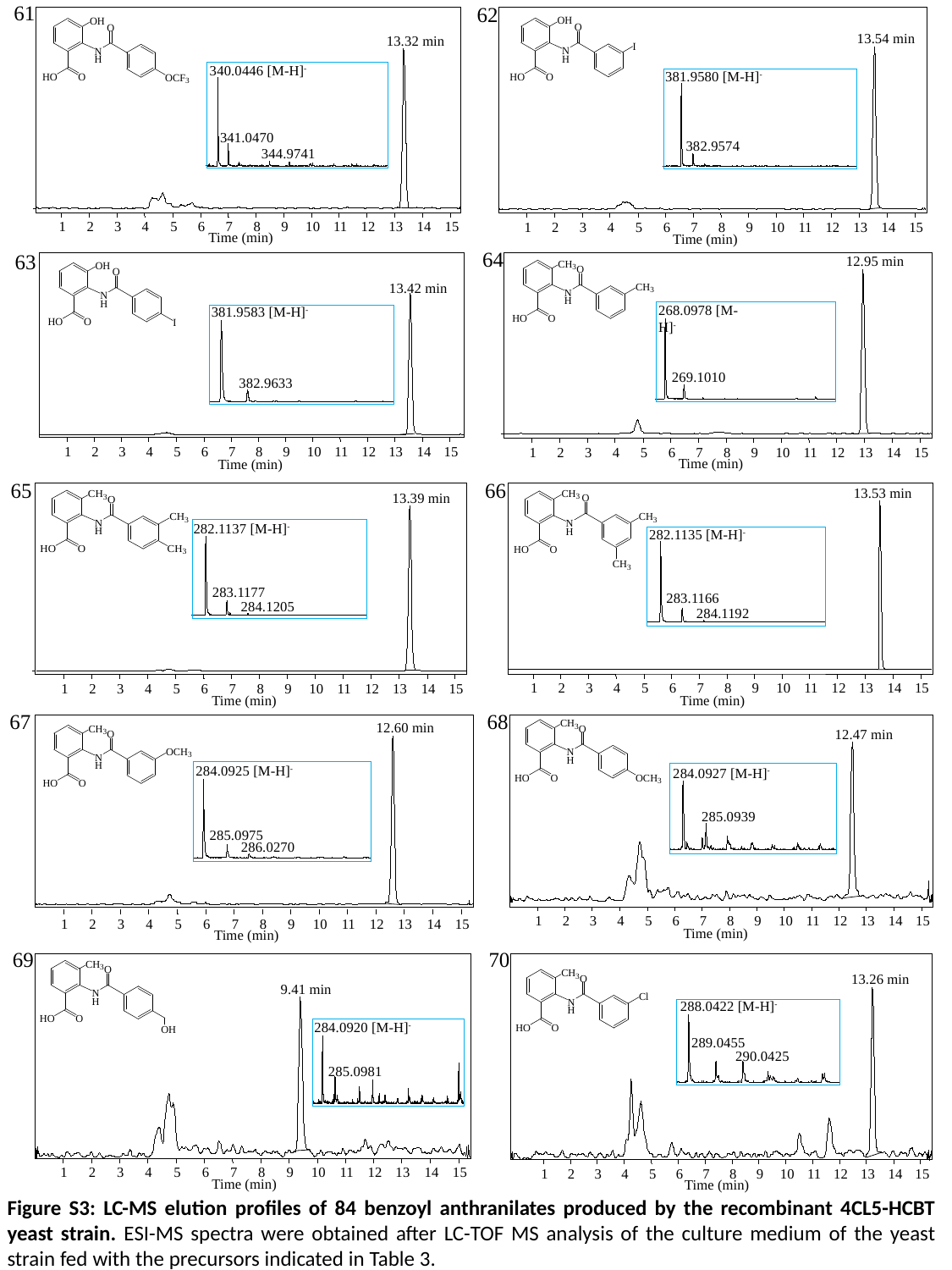

61
62
13.32 min
340.0446 [M-H]-
341.0470
344.9741
1
2
3
4
5
6
7
8
9
10
11
12
13
14
15
Time (min)
13.54 min
381.9580 [M-H]-
382.9574
1
2
3
4
5
6
7
8
9
10
11
12
13
14
15
Time (min)
64
63
12.95 min
13.42 min
381.9583 [M-H]-
382.9633
1
2
3
4
5
6
7
8
9
10
11
12
13
14
15
Time (min)
268.0978 [M-H]-
269.1010
1
2
3
4
5
6
7
8
9
10
11
12
13
14
15
Time (min)
65
66
13.39 min
282.1137 [M-H]-
283.1177
284.1205
1
2
3
4
5
6
7
8
9
10
11
12
13
14
15
Time (min)
13.53 min
282.1135 [M-H]-
283.1166
284.1192
1
2
3
4
5
6
7
8
9
10
11
12
13
14
15
Time (min)
67
68
12.47 min
284.0927 [M-H]-
285.0939
1
2
3
4
5
6
7
8
9
10
11
12
13
14
15
Time (min)
12.60 min
284.0925 [M-H]-
285.0975
286.0270
1
2
3
4
5
6
7
8
9
10
11
12
13
14
15
Time (min)
69
70
9.41 min
284.0920 [M-H]-
285.0981
1
2
3
4
5
6
7
8
9
10
11
12
13
14
15
Time (min)
13.26 min
288.0422 [M-H]-
289.0455
290.0425
1
2
3
4
5
6
7
8
9
10
11
12
13
14
15
Time (min)
Figure S3: LC-MS elution profiles of 84 benzoyl anthranilates produced by the recombinant 4CL5-HCBT yeast strain. ESI-MS spectra were obtained after LC-TOF MS analysis of the culture medium of the yeast strain fed with the precursors indicated in Table 3.

## Slide 8
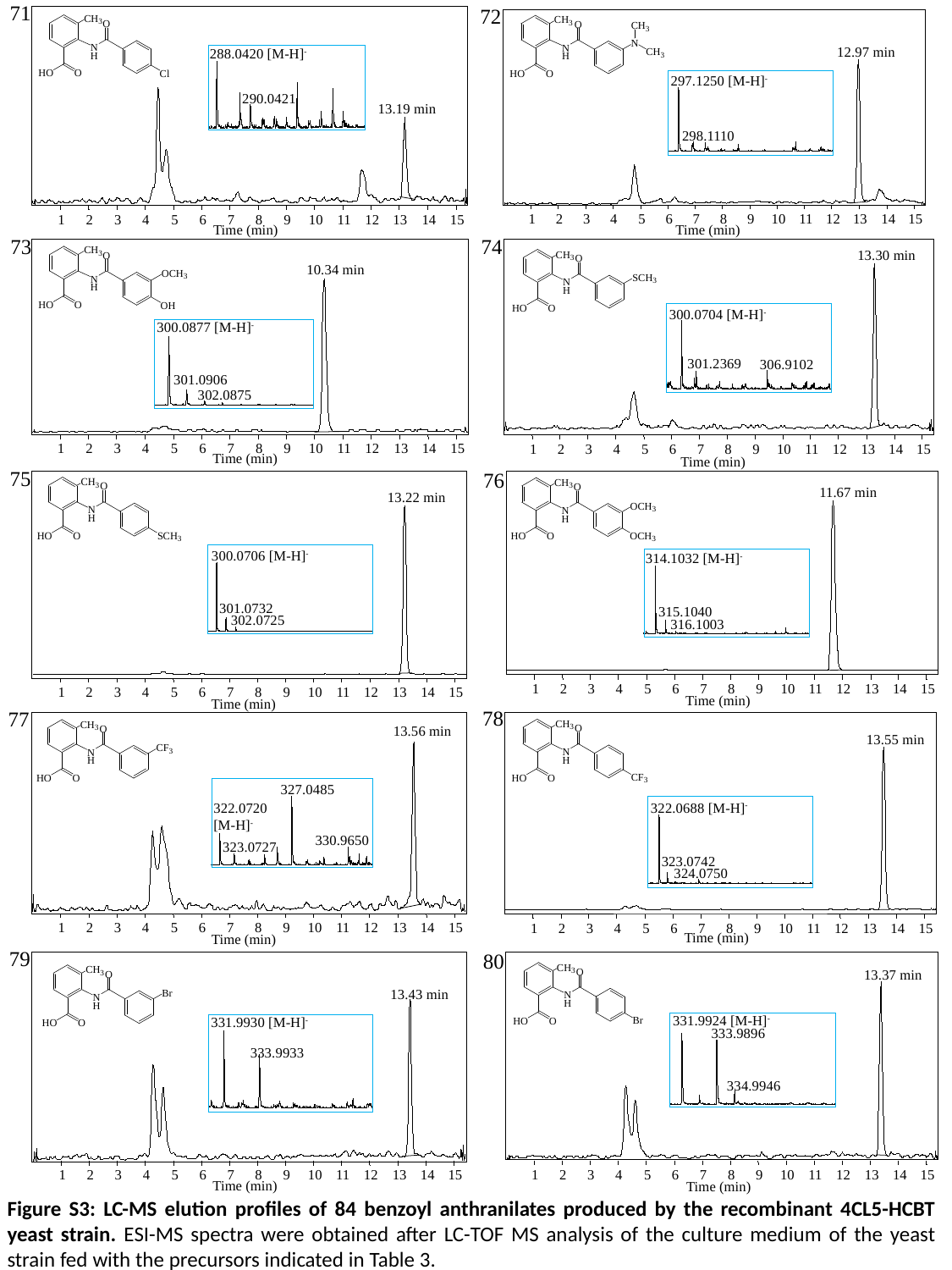

71
72
288.0420 [M-H]-
290.0421
13.19 min
1
2
3
4
5
6
7
8
9
10
11
12
13
14
15
Time (min)
12.97 min
297.1250 [M-H]-
298.1110
1
2
3
4
5
6
7
8
9
10
11
12
13
14
15
Time (min)
73
74
10.34 min
300.0877 [M-H]-
301.0906
302.0875
1
2
3
4
5
6
7
8
9
10
11
12
13
14
15
Time (min)
13.30 min
300.0704 [M-H]-
301.2369
306.9102
1
2
3
4
5
6
7
8
9
10
11
12
13
14
15
Time (min)
75
76
13.22 min
300.0706 [M-H]-
301.0732
302.0725
1
2
3
4
5
6
7
8
9
10
11
12
13
14
15
Time (min)
 11.67 min
314.1032 [M-H]-
315.1040
316.1003
1
2
3
4
5
6
7
8
9
10
11
12
13
14
15
Time (min)
78
77
13.56 min
327.0485
322.0720
[M-H]-
330.9650
323.0727
1
2
3
4
5
6
7
8
9
10
11
12
13
14
15
Time (min)
13.55 min
322.0688 [M-H]-
323.0742
324.0750
1
2
3
4
5
6
7
8
9
10
11
12
13
14
15
Time (min)
79
80
13.43 min
331.9930 [M-H]-
333.9933
1
2
3
4
5
6
7
8
9
10
11
12
13
14
15
Time (min)
13.37 min
331.9924 [M-H]-
333.9896
334.9946
1
2
3
4
5
6
7
8
9
10
11
12
13
14
15
Time (min)
Figure S3: LC-MS elution profiles of 84 benzoyl anthranilates produced by the recombinant 4CL5-HCBT yeast strain. ESI-MS spectra were obtained after LC-TOF MS analysis of the culture medium of the yeast strain fed with the precursors indicated in Table 3.

## Slide 9
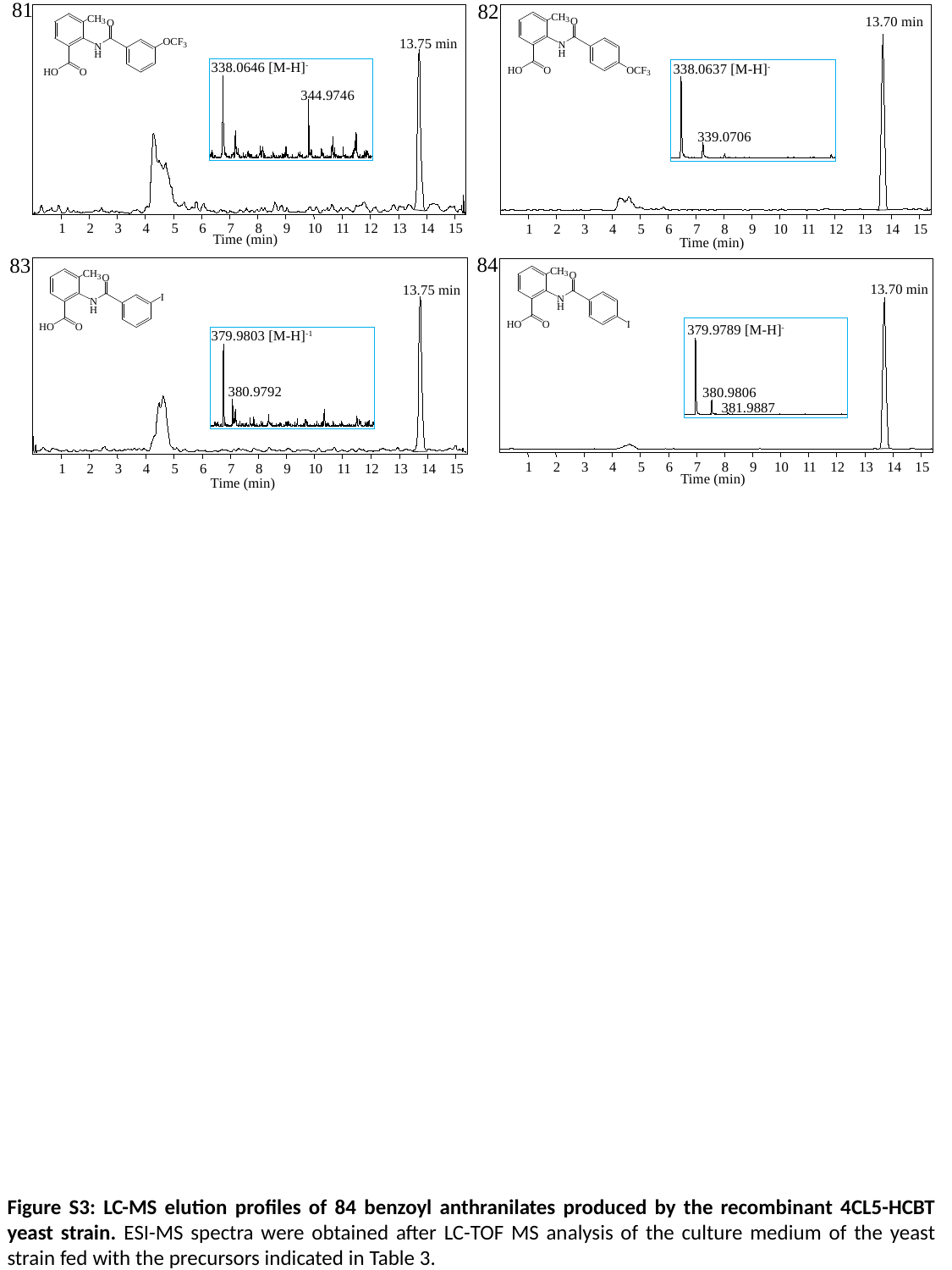

81
82
13.75 min
338.0646 [M-H]-
344.9746
1
2
3
4
5
6
7
8
9
10
11
12
13
14
15
Time (min)
13.70 min
338.0637 [M-H]-
339.0706
1
2
3
4
5
6
7
8
9
10
11
12
13
14
15
Time (min)
84
83
13.75 min
379.9803 [M-H]-1
380.9792
1
2
3
4
5
6
7
8
9
10
11
12
13
14
15
Time (min)
 13.70 min
379.9789 [M-H]-
380.9806
381.9887
1
2
3
4
5
6
7
8
9
10
11
12
13
14
15
Time (min)
Figure S3: LC-MS elution profiles of 84 benzoyl anthranilates produced by the recombinant 4CL5-HCBT yeast strain. ESI-MS spectra were obtained after LC-TOF MS analysis of the culture medium of the yeast strain fed with the precursors indicated in Table 3.
