# Supplementary material for: Precursor-Directed Combinatorial Biosynthesis of Cinnamoyl, Dihydrocinnamoyl, and Benzoyl Anthranilates in Saccharomyces cerevisiae
Source: PLoS One. 2015 Oct 2;10(10):e0138972. doi: 10.1371/journal.pone.0138972 (PMC4591981; doi:10.1371/journal.pone.0138972)
Supplement: S1 Table — (DOCX) [file pone.0138972.s004.docx]

| **Table S1.** Structures and concentrations of the cinnamates used for the yeast feedings. | | | | | |
| --- | --- | --- | --- | --- | --- |
| **Cinnamate donors tested**  | **R_3_** | **R_4_** | **R_5_** | **R_6_** | **Concentration**  **(µM)** |
| *p*-coumaric acid | H | H | OH | H | 300 |
| 2-fluorocinnamic acid | F | H | H | H | 50 |
| 3-fluorocinnamic acid | H | F | H | H | 50 |
| 4-fluorocinnamic acid | H | H | F | H | 50 |
| 2-chlorocinnamic acid | Cl | H | H | H | 50 |
| 4-chlorocinnamic acid | H | H | Cl | H | 50 |
| 2-bromocinnamic acid | Br | H | H | H | 50 |
| 3-bromocinnamic acid | H | Br | H | H | 50 |
| 4-bromocinnamic acid | H | H | Br | H | 15 |
| 2-trifluoromethylcinnamic acid | CF_3_ | H | H | H | 50 |
| 3-trifluoromethylcinnamic acid | H | CF_3_ | H | H | 25 |
| 4-trifluoromethylcinnamic acid | H | H | CF_3_ | H | 15 |
| 3-difluoromethoxycinnamic acid | H | OCHF_2_ | H | H | 50 |
| 4-difluoromethoxycinnamic acid | H | H | OCHF2 | H | 50 |
| 3-trifluoromethoxycinnamic acid | H | OCF_3_ | H | H | 50 |
