# Supplementary material for: Precursor-Directed Combinatorial Biosynthesis of Cinnamoyl, Dihydrocinnamoyl, and Benzoyl Anthranilates in Saccharomyces cerevisiae
Source: PLoS One. 2015 Oct 2;10(10):e0138972. doi: 10.1371/journal.pone.0138972 (PMC4591981; doi:10.1371/journal.pone.0138972)
Supplement: S2 Table — (DOCX) [file pone.0138972.s005.docx]

| **Table S2.** Structures and concentrations of the dihydrocinnamates used for the yeast feedings. | | | | | |
| --- | --- | --- | --- | --- | --- |
| **Dihydrocinnamate donors tested** | **R_3_** | **R_4_** | **R_5_** | **R_6_** | **Concentration**  **(µM)** |
| 3,4-dihydroxydihydrocinnamic acid | H | OH | OH | H | 300 |
| 3-methoxydihydrocinnamic acid | H | OCH_3_ | H | H | 300 |
| 2-hydroxydihydrocinnamic acid | OH | H | H | H | 1000 |
| 3-hydroxydihydrocinnamic acid | H | OH | H | H | 300 |
| 2-methoxydihydrocinnamic acid | OCH_3_ | H | H | H | 300 |
| 3-methoxy-4-hydroxydihydrocinnamic acid | H | OCH_3_ | OH | H | 300 |
| 2,5-dimethoxydihydrocinnamic acid | OCH_3_ | H | OH | OCH_3_ | 300 |
| 3-fluorodihydrocinnamic acid | H | F | H | H | 300 |
| 4-fluorodihydrocinnamic acid | H | H | F | H | 300 |
| 3-chlorodihydrocinnamic acid | H | Cl | H | H | 300 |
| 4-hydroxydihydrocinnamic acid | H | H | OH | H | 300 |
| dihydrocinnamic acid | H | H | H | H | 300 |
| 4-methyldihydrocinnamic acid | H | H | CH_3_ | H | 50 |
| 3-methyldihydrocinnamic acid | H | CH_3_ | H | H | 50 |
| 3,5-dimethoxy-4-hydroxydihydrocinnamic acid | H | OCH_3_ | OH | OCH_3_ | 50 |
| 2,3-dimethoxydihydrocinnamic acid | OCH_3_ | OCH_3_ | H | H | 300 |
| 2,4-dimethoxydihydrocinnamic acid | OCH_3_ | H | OCH_3_ | H | 300 |
| 3,4-dimethoxydihydrocinnamic acid | H | OCH_3_ | OCH_3_ | H | 300 |
| 4-methoxydihydrocinnamic acid | H | H | OCH_3_ | H | 300 |
| 2-fluorodihydrocinnamic acid | F | H | H | H | 300 |
| 2-methyldihydrocinnamic acid | CH_3_ | H | H | H | 300 |
| 3-difluoromethoxydihydrocinnamic acid | H | OCHF_2_ | H | H | 50 |
| 4-difluoromethoxydihydrocinnamic acid | H | H | OCHF_2_ | H | 50 |
|  |  |  |  |  |  |
|  |  |  |  |  |  |
