# Supplementary material for: Precursor-Directed Combinatorial Biosynthesis of Cinnamoyl, Dihydrocinnamoyl, and Benzoyl Anthranilates in Saccharomyces cerevisiae
Source: PLoS One. 2015 Oct 2;10(10):e0138972. doi: 10.1371/journal.pone.0138972 (PMC4591981; doi:10.1371/journal.pone.0138972)
Supplement: S3 Table — (DOCX) [file pone.0138972.s006.docx]

| **Table S3**. Structures and concentrations of the benzoates used for the yeast feedings. | | | | | | |
| --- | --- | --- | --- | --- | --- | --- |
| **Benzoate donors tested** | **R_3_** | **R_4_** | **R_5_** | **R_6_** | **R_7_** | **Concentration**  **(µM)** |
| 3-chlorobenzoic acid  | H | Cl | H | H | H | 50 |
| 4-hydroxybenzoic acid | H | H | OH | H | H | 300 |
| 2-fluorobenzoic acid | F | H | H | H | H | 500 |
| 3-fluorobenzoic acid | H | F | H | H | H | 100 |
| 4-fluorobenzoic acid | H | H | F | H | H | 100 |
| 3-methoxybenzoic acid | H | OCH_3_ | H | H | H | 300 |
| 4-methoxybenzoic acid | H | H | OCH_3_ | H | H | 300 |
| 3-hydroxybenzoic acid | H | OH | H | H | H | 1000 |
| 3-bromobenzoic acid | H | Br | H | H | H | 50 |
| 2-chlorobenzoic acid | Cl | H | H | H | H | 300 |
| 4-chlorobenzoic acid | H | H | Cl | H | H | 100 |
| 2-methylbenzoic acid | CH_3_ | H | H | H | H | 300 |
| 4-methylbenzoic acid | H | H | CH_3_ | H | H | 50 |
| 3-aminobenzoic acid | H | NH_2_ | H | H | H | 1000 |
| 3-methylthiobenzoic acid | H | SCH_3_ | H | H | H | 50 |
| 3-trifluoromethoxybenzoic acid | H | OCF_3_ | H | H | H | 50 |
| 3-methylbenzoic acid | H | CH_3_ | H | H | H | 300 |
| 4-nitrobenzoic acid | H | H | NO_2_ | H | H | 50 |
| 4-trifluoromethoxybenzoic acid | H | H | OCF_3_ | H | H | 35 |
| 2-amino-3-hydroxybenzoic acid | NH_2_ | OH | H | H | H | 300 |
| 3-dimethylaminobenzoic acid | H | N(CH_3_)_2_ | H | H | H | 300 |
| 4-dimethylaminobenzoic acid | H | N(CH_3_)_2_ | H | H | H | 300 |
| 4-hydroxymethylbenzoic acid | H | H | CH_2_OH | H | H | 300 |
| 2-amino-5-methylbenzoic acid | NH_2_ | H | H | CH_3_ | H | 300 |
| 3-methoxy-4-hydroxybenzoic acid | H | OCH_3_ | OH | H | H | 300 |
| benzoic acid | H | H | H | H | H | 300 |
| 3-trifluoromethylbenzoic acid | H | CF_3_ | H | H | H | 50 |
| 4-trifluoromethylbenzoic acid | H | H | CF_3_ | H | H | 15 |
| 2-amino-5-methylbenzoic acid | NH_2_ | H | H | CH_3_ | H | 300 |
| 3,4-dimethoxybenzoic acid | H | OCH_3_ | OCH_3_ | H | H | 300 |
| 2,5-dimethylbenzoic acid | CH_3_ | H | H | CH_3_ | H | 300 |
| 3,4-dimethylbenzoic acid | H | CH_3_ | CH_3_ | H | H | 300 |
| 3,5-dimethylbenzoic acid | H | CH_3_ | H | CH_3_ | H | 300 |
| 3-iodobenzoic acid | H | I | H | H | H | 15 |
| 4-iodobenzoic acid | H | H | I | H | H | 15 |
| 2-hydroxybenzoic acid | OH | H | H | H | H | 300 |
| 3,4-dihydroxybenzoic acid | H | OH | OH | H | H | 300 |
| 4-hydroxy-3,5-dimethoxybenzoic acid | H | OCH_3_ | OH | OCH_3_ | H | 300 |
| 3,4,5-trihydroxybenzoic acid | H | OH | OH | OH | H | 300 |
| 2,6-dihydroxybenzoic acid | OH | H | H | H | OH | 300 |
| 3,5-dihydroxybenzoic acid | H | OH | H | OH | H | 300 |
| 2,3-dihydroxybenzoic acid | OH | OH | H | H | H | 300 |
| 2,5-dihydroxybenzoic acid | OH | H | H | OH | H | 300 |
| 2,4-dihydroxybenzoic acid | OH | H | OH | H | H | 300 |
| 2-methoxybenzoic acid | OCH_3_ | H | H | H | H | 300 |
| 2-bromobenzoic acid | Br | H | H | H | H | 300 |
| 4-bromobenzoic acid | H | H | Br | H | H | 15 |
| 2-iodobenzoic acid | I | H | H | H | H | 300 |
| 2-nitrobenzoic acid | NO_2_ | H | H | H | H | 300 |
| 3-nitrobenzoic acid | H | NO_2_ | H | H | H | 300 |
| 4-aminobenzoic acid | H | H | NH_2_ | H | H | 300 |
| 2-trifluoromethoxybenzoic acid | OCF_3_ | H | H | H | H | 300 |
| 2-aminobenzoic acid | NH_2_ | H | H | H | H | 500 |
| 2-amino-3-methoxybenzoic acid | NH_2_ | OCH_3_ | H | H | H | 300 |
| 2-trifluoromethylbenzoic acid | CF_3_ | H | H | H | H | 300 |
| 2-amino-5-hydroxybenzoic acid | NH_2_ | H | H | OH | H | 300 |
| 2,6-difluorobenzoic acid | F | H | H | H | F | 300 |
| 2,6-dimethoxybenzoic acid | OCH_3_ | H | H | H | OCH_3_ | 300 |
| 2,5-dimethoxybenzoic acid | OCH_3_ | H | H | OCH_3_ | H | 300 |
| 4-methylthiobenzoic acid | H | H | SCH_3_ | H | H | 15 |
| 2-amino-5-chlorobenzoic acid | NH_2_ | H | H | Cl | H | 50 |
| 2-amino-5-nitrobenzoic acid | NH_2_ | H | H | NO_2_ | H | 300 |
| 2-amino-5-iodobenzoic acid | NH_2_ | H | H | I | H | 50 |
| 2-amino-3-trifluoromethylbenzoic acid | NH_2_ | CF_3_ | H | H | H | 50 |
| 3,5-dimethoxybenzoic acid | H | OCH_3_ | H | OCH_3_ | H | 300 |
| 2,4-dimethoxybenzoic acid | OCH_3_ | H | OCH_3_ | H | H | 50 |
| 2,3-dimethoxybenzoic acid | OCH_3_ | OCH_3_ | H | H | H | 300 |
| 2-amino-3-chlorobenzoic acid | NH_2_ | Cl | H | H | H | 50 |
| 2,3-dimethylbenzoic acid | CH_3_ | CH_3_ | H | H | H | 300 |
| 2,6-dimethylbenzoic acid | CH_3_ | H | H | H | CH_3_ | 300 |
| 2,4-dimethylbenzoic acid | CH_3_ | H | CH_3_ | H | H | 300 |
| 2-amino-5-fluorobenzoic acid | NH_2_ | H | H | F | H | 300 |
| 2-phenoxybenzoic acid | OPh | H | H | H | H | 300 |
| 3-phenoxybenzoic acid | H | OPh | H | H | H | 50 |
| 4-phenoxybenzoic acid | H | H | OPh | H | H | 300 |
